# Integrating Artificial Intelligence into Perinatal Care Pathways: A Scoping Review of Reviews of Applications, Outcomes, and Equity

**DOI:** 10.3390/nursrep15080281

**Published:** 2025-07-31

**Authors:** Rabie Adel El Arab, Omayma Abdulaziz Al Moosa, Zahraa Albahrani, Israa Alkhalil, Joel Somerville, Fuad Abuadas

**Affiliations:** 1 Almoosa College of Health Sciences, Alhsa 36422, Saudi Arabia; 2 Almoosa Specialist Hospital, Alhsa 36342, Saudi Arabia; 3 Inverness College, University of the Highlands and Islands, Inverness IV2 3JH, UK; joel.somerville@uhi.ac.uk; 4 Department of Community Health Nursing, College of Nursing, Jouf University, Sakakah 72388, Saudi Arabia; fhabuadas@ju.edu.sa

**Keywords:** artificial intelligence, machine learning, nursing, perinatal care, maternal health, neonatal care, reproductive health, health equity

## Abstract

**Background:** Artificial intelligence (AI) and machine learning (ML) have been reshaping maternal, fetal, neonatal, and reproductive healthcare by enhancing risk prediction, diagnostic accuracy, and operational efficiency across the perinatal continuum. However, no comprehensive synthesis has yet been published. **Objective:** To conduct a scoping review of reviews of AI/ML applications spanning reproductive, prenatal, postpartum, neonatal, and early child-development care. **Methods:** We searched PubMed, Embase, the Cochrane Library, Web of Science, and Scopus through April 2025. Two reviewers independently screened records, extracted data, and assessed methodological quality using AMSTAR 2 for systematic reviews, ROBIS for bias assessment, SANRA for narrative reviews, and JBI guidance for scoping reviews. **Results:** Thirty-nine reviews met our inclusion criteria. In preconception and fertility treatment, convolutional neural network-based platforms can identify viable embryos and key sperm parameters with over 90 percent accuracy, and machine-learning models can personalize follicle-stimulating hormone regimens to boost mature oocyte yield while reducing overall medication use. Digital sexual-health chatbots have enhanced patient education, pre-exposure prophylaxis adherence, and safer sexual behaviors, although data-privacy safeguards and bias mitigation remain priorities. During pregnancy, advanced deep-learning models can segment fetal anatomy on ultrasound images with more than 90 percent overlap compared to expert annotations and can detect anomalies with sensitivity exceeding 93 percent. Predictive biometric tools can estimate gestational age within one week with accuracy and fetal weight within approximately 190 g. In the postpartum period, AI-driven decision-support systems and conversational agents can facilitate early screening for depression and can guide follow-up care. Wearable sensors enable remote monitoring of maternal blood pressure and heart rate to support timely clinical intervention. Within neonatal care, the Heart Rate Observation (HeRO) system has reduced mortality among very low-birth-weight infants by roughly 20 percent, and additional AI models can predict neonatal sepsis, retinopathy of prematurity, and necrotizing enterocolitis with area-under-the-curve values above 0.80. From an operational standpoint, automated ultrasound workflows deliver biometric measurements at about 14 milliseconds per frame, and dynamic scheduling in IVF laboratories lowers staff workload and per-cycle costs. Home-monitoring platforms for pregnant women are associated with 7–11 percent reductions in maternal mortality and preeclampsia incidence. Despite these advances, most evidence derives from retrospective, single-center studies with limited external validation. Low-resource settings, especially in Sub-Saharan Africa, remain under-represented, and few AI solutions are fully embedded in electronic health records. **Conclusions:** AI holds transformative promise for perinatal care but will require prospective multicenter validation, equity-centered design, robust governance, transparent fairness audits, and seamless electronic health record integration to translate these innovations into routine practice and improve maternal and neonatal outcomes.

## 1. Introduction

Artificial intelligence (AI) and machine learning (ML) have expanded rapidly in maternal, fetal, neonatal, and reproductive healthcare, promising advances in risk prediction, diagnostics, and treatment optimization. AI/ML use cases from three thematic domains: Predictive analytics: models for preeclampsia, gestational diabetes, preterm birth, and mode-of-delivery forecasting (AUCs up to 0.99). Diagnostic imaging: automated ultrasound segmentation (Dice > 0.90), anomaly detection with explainable ResNet/transformer models (sensitivity > 93%), and biometric tools estimating gestational age within one week and fetal weight within ~190 g. Operational efficiency: workflow automation (≈14 ms/frame for fetal biometry), IVF laboratory scheduling optimizers, and remote monitoring platforms linked to 7–11% reductions in maternal mortality and preeclampsia. Recent systematic reviews report high performance of AI models—for example, obstetrics and midwifery AI models have achieved high accuracy in assisted reproduction, imaging diagnosis, pregnancy risk assessment (e.g., preeclampsia, gestational diabetes, preterm birth), fetal monitoring, mode-of-birth prediction, and perinatal outcomes [1]. Similarly, AI-driven models in neonatal care show the potential to improve monitoring and prognostication [2].

AI applications in maternal healthcare include risk prediction, diagnostics, and decision support during pregnancy and childbirth. ML models have been applied to predict preeclampsia, preterm birth, gestational diabetes, hemorrhaging, and delivery mode using maternal demographic, clinical, and laboratory data. For example, a systematic review of four ML-based studies on preeclampsia reported AUC values ranging from 0.860 to 0.973 [3], indicating a strong predictive performance. These AUC estimates derive from retrospective, single-center cohorts (*n* = 120–350) and lack reported confidence intervals, limiting generalizability. Similarly, ML models for preterm birth prediction in diverse datasets show potential but are limited by inconsistent reporting and unvalidated methodology [4]. A systematic review of mode-of-delivery studies found that AI models outperformed traditional statistical approaches in forecasting cesarean versus vaginal birth, using predictors such as maternal age, parity, gestational age, labor interventions, and fetal weight [5].

Other maternal use cases include automated analysis of cardiotocography (fetal heart rate monitoring) and ultrasound imaging. AI tools can interpret electronic fetal monitoring patterns to detect distress and can measure fetal biometry more objectively in routine ultrasounds. A recent review notes that integrating AI into maternal care could enhance workflow by shortening exam time and reducing clinician workload [6]. However, formal cost-effectiveness and budget-impact analyses are scarce, precluding firm conclusions about economic benefits. For example, deep learning models can automatically locate standard ultrasound views and compute head circumference or abdominal diameter, which may improve consistency and free clinicians for higher-level tasks. Additionally, AI is being applied to fetal assessment, particularly in prenatal imaging and anomaly detection [6]. In congenital heart disease screening, AI models have demonstrated the ability to detect complex heart lesions that can be difficult for sonographers to recognize quickly [6].

Fetal monitoring is another major application. AI algorithms analyze continuous fetal heart rate patterns (cardiotocography) to predict hypoxia or distress with high accuracy [7].

In the neonatal period, AI-driven decision support targets NICU/PICU care and neonatal outcomes. Systematic reviews indicate many AI models for neonatal risk assessment and monitoring (e.g., infection, respiratory failure, hypoglycemia, retinopathy of prematurity) have been developed, but few have progressed to clinical use [2]. Notably, only ~16% of published AI algorithms have undergone external multicenter validation or secured regulatory approval. This confirms that nearly all neonatal AI research is still in testing phases.

AI also shows promise in assisted reproductive technology (ART). A recent systematic review found that AI significantly enhances ART processes, improving efficiency: streamlined workflows and better patient selection could shorten treatment cycles and reduce costs [8].

Real-world implementation of AI in health care is hampered by three core challenges: first, data and system issues—electronic health records and imaging are often incomplete, noisy, and stored in non-interoperable silos, undermining model reliability; second, algorithmic limitations—biases in training data, fairness concerns, and opaque “black-box” decision processes erode clinician trust and risk perpetuating health disparities; and third, operational and regulatory constraints based on geographic distribution and equity [9]. Transfer-learning experiments reveal performance drops in low-resource or ethnically diverse cohorts, and formal fairness audits are rarely conducted, raising equity concerns. Regulatory pathways for adaptive, continuously learning AI systems remain undefined in most jurisdictions, further impeding clinical translation.

Beyond maternal–fetal–neonatal care, integrative analyses in adjacent disciplines illustrate AI’s real-world scalability in low-resource settings. For example, Hassanein et al., (2025) [10] demonstrated that wearable sensor-driven early warning systems and automated alert platforms can markedly improve workflow efficiency and reduce staff burnout in nursing units—approaches readily adaptable to maternity and neonatal wards with constrained staffing and infrastructure. Additionally, there is a critical need for AI literacy and robust ethical frameworks to prepare frontline clinicians for safe, equitable adoption across diverse care environments [11,12].

Collectively, the existing reviews paint a picture of immense AI promise but limited evidence of impact. Many systematic reviews note high algorithmic accuracy and potential applications, yet few note successful implementation. These fragmentary findings underscore the need for a scoping review of reviews. By aggregating evidence from multiple reviews, a scoping synthesis can more comprehensively assess what is known (and unknown) about AI in perinatal health.

Aim:

To comprehensively synthesize evidence from existing systematic and narrative reviews on artificial intelligence applications across maternal, fetal, neonatal, and reproductive healthcare—encompassing model performance, clinical and economic outcomes, implementation barriers, and ethical–regulatory considerations.

Objectives:

1. To catalog and characterize key AI use cases—ranging from maternal complication risk prediction and tele-monitoring, through fetal anomaly detection and gestational age estimation, to neonatal intensive-care decision support and assisted-reproduction workflow optimization—across the continuum of maternal, fetal, neonatal, and reproductive health.
2. To assess diagnostic performance metrics (e.g., AUC, Dice), and—where reported—patient-centered outcomes, workflow-efficiency measures, and clinician-time savings.
3. To summarize evidence on AI’s effects on operational metrics—such as scan-time reductions, staff time savings, cost-effectiveness, and automation-driven throughput gains.
4. To systematically identify and assess the hurdles to clinical implementation.

## 2. Methods

### 2.1. Study Design

A scoping review of reviews [13] that synthesizes findings from systematic, scoping, and narrative reviews of AI/ML applications in reproductive, prenatal, postpartum, neonatal, and early child-development care. We followed the PRISMA-ScR guidelines [14] to ensure a rigorous and transparent approach. The review process adhered to a structured methodology encompassing eligibility criteria, search strategy, screening and selection, data extraction, synthesis, and bias assessment. Moreover, most reviews did not report dual-reviewer screening or formal publication-bias assessments, increasing the risk of selective reporting. A thematic synthesis approach [15] was chosen to structure and interpret the findings, as it allows for the identification and organization of recurring patterns and themes across diverse datasets.

### 2.2. Spider Framework for Eligibility

We adopted the Sample, Phenomenon of Interest, Design, Evaluation, Research type (SPIDER) tool to define eligibility for evidence syntheses [16]. We applied the SPIDER framework [13] to define eligibility for this scoping review of reviews, explicitly to capture both quantitative evidence (model performance, efficiency metrics, economic outcomes) and qualitative insights (implementation barriers, equity analyses, ethical/regulatory themes). While PRISMA-ScR guided our reporting, SPIDER was selected to define eligibility for both qualitative and quantitative syntheses, ensuring comprehensive capture of diverse review designs. The sample comprised reviews—systematic (with or without meta-analysis), scoping, or narrative—that examined AI or machine-learning methods in human reproductive, prenatal, postpartum, neonatal, or early-child-development settings. The phenomenon of interest covered AI-driven algorithms (including machine learning, deep learning, neural networks, and ensemble approaches) applied to diagnostic, predictive, or workflow optimization. The design encompassed evidence syntheses published in peer-reviewed journals. The evaluation referred to reported metrics, such as area under the curve, sensitivity, specificity, workflow time savings, economic outcomes, or equity analyses. The research types included both quantitative and qualitative reviews. (Table 1: SPIDER Framework). We integrated systematic, scoping, and narrative reviews to leverage their complementary strengths: systematic reviews for quantitative effect estimates, scoping reviews for mapping emerging topics, and narrative reviews for contextual interpretation. Findings were explicitly weighted (highest certainty to systematic, moderate to scoping, supportive to narrative) to manage heterogeneity across review types.

### 2.3. Search Strategy and Information Sources

**Figure 1:**
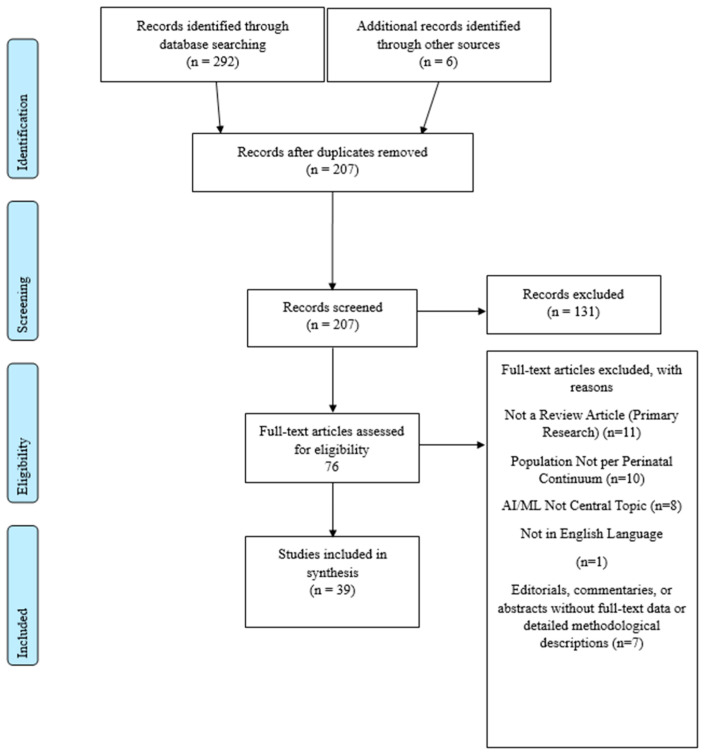
PRISMA-ScR Flow Diagram.

From inception to April 2025, we conducted parallel searches in five databases: MEDLINE (via PubMed), EMBASE, Cochrane Database of Systematic Reviews, Web of Science, and CINAHL. Search strategies combined controlled vocabularies (e.g., “Artificial Intelligence” [Mesh], “Machine Learning” [Mesh]) with free-text terms (“deep learning,” “neural network*,” “in vitro fertilization,” “fetal monitoring,” “NICU”). A MEDLINE strategy is shown in Table 2; equivalent syntax was adapted for each database. Hand searches of reference lists and key journals supplemented the electronic retrieval. A detailed PRISMA-ScR flow diagram (Figure 1) is provided to transparently report the screening and selection process.

### 2.4. Inclusion and Exclusion Criteria

From inception through April 2025, we included peer-reviewed systematic, scoping, or narrative reviews of AI and ML applications in reproductive, prenatal, postpartum, neonatal, and early child-development care that reported at least one of the following outcomes: diagnostic performance, workflow efficiency, economic measures, or equity analyses. We excluded primary research studies, review protocols, editorials, letters, conference abstracts, non-English publications, animal or in vitro research, and any reviews lacking an explicit focus on AI/ML.

### 2.5. Selection Process

The records identified through database searches were imported into Rayyan, a systematic review screening tool [17]. Two reviewers (RA, FA) independently screened all titles, abstracts, and full texts. The inter-rater agreement was excellent (Cohen’s κ = 0.82 for title/abstract screening; κ = 0.87 for full-text eligibility), and discrepancies were resolved by consensus. Data extraction achieved 94% raw agreement, with remaining disagreements adjudicated by a third reviewer (JS).

### 2.6. Data Extraction and Synthesis

For each review, we recorded the citation details (authors, year, review type, and scope) and delineated the AI application domain, such as preconception care, fetal monitoring, postpartum wellness, or neonatal decision support. Technical aspects—including model architectures (convolutional neural networks, recurrent neural networks, transformer architectures, ensemble algorithms) and input modalities (imaging, genomic data, wearable sensors, electronic health records)—were mapped alongside quantitative performance indicators (area under the receiver operating characteristic curve, sensitivity, specificity, Dice coefficients, and mean absolute error). We further documented operational outcomes, such as reductions in processing time and improvements in throughput, and economic measures, including cost savings and resource utilization metrics. Validation parameters were extracted, specifying whether reviews included retrospective or prospective designs, single- or multicenter cohorts, and the extent of external validation. Equity considerations encompassed geographic distribution, representation of low-resource settings, and any subgroup or fairness analyses. Finally, we noted implementation and governance challenges, including data quality and availability, system integration difficulties, model interpretability constraints, and ethical or regulatory issues, such as data privacy, consent frameworks, and the status of clinical approval pathways. We integrated systematic, scoping, and narrative reviews to leverage the complementary strengths of each design—systematic reviews for quantitative effect estimates, scoping reviews for breadth of emerging topics, and narrative reviews for contextual interpretation. We explicitly weighted findings by review type (assigning highest certainty to systematic reviews, moderate certainty to scoping reviews, and supportive insights from narrative reviews) to ensure balanced synthesis across heterogeneous evidence.

### 2.7. Assessment of Methodological Quality

We selected AMSTAR 2 (A Measurement Tool to Assess Systematic Reviews 2) [18] as our principal quality-assessment instrument because it is the most comprehensive, validated framework for critically appraising a wide spectrum of evidence syntheses, encompassing both meta-analytic and non-meta-analytic reviews. AMSTAR 2’s 16 domains interrogate core methodological conduct (e.g., protocol registration, search strategy, duplicate processes, risk-of-bias appraisal) and reporting transparency, explicitly flagging critical deficiencies—such as absent protocols, incomplete searches, lack of independent screening/extraction, and failure to evaluate primary-study bias—that can compromise review credibility. Two reviewers (ZA, SA) independently applied AMSTAR 2’s critical domains—protocol registration, comprehensive searching, duplicate selection and extraction, listing of excluded and included studies, formal risk-of-bias appraisal, and publication-bias investigation—to each review. To respect methodological differences, all 16 domains were assessed for systematic reviews, whereas narrative and scoping reviews were scored only on domains relevant to transparent reporting, with domains requiring formal systematic methods marked “Not Applicable” in accordance with JBI scoping guidance. Discrepancies between reviewers were first addressed through consensus meetings; any remaining disagreements were adjudicated by a third reviewer (OA, JS).

### 2.8. Risk-of-Bias

To delineate specific bias threats beyond the aggregate confidence provided by AMSTAR 2, we applied complementary, domain-focused instruments according to review type.

We employed three tools, the first is ROBIS for systematic reviews [19]—a rigorously developed tool for assessing risk of bias in systematic reviews—in three structured phases. In Phase 1 (Relevance), each review’s scope and objectives were confirmed to align with our review question (screening only). In Phase 2 (Domain Assessment), two independent reviewers (RA, FA) evaluated four domains via standardized signaling questions: (1) Eligibility Criteria—transparency and appropriateness of inclusion/exclusion rules; (2) Identification and Selection of Studies—comprehensiveness of search strategy and screening processes; (3) Data Collection and Appraisal—rigor of data extraction methods and formal critical appraisal of primary studies; and (4) Synthesis and Findings—appropriateness of synthesis methods, including exploration of heterogeneity and sensitivity analyses. Each domain was rated as “low,” “high,” or “unclear” risk. In Phase 3 (Overall Judgment), an overarching risk-of-bias rating was assigned, with any serious domain-level concern resulting in a “high-risk” classification. Discrepancies were resolved by consensus or, if required, adjudication by a third reviewer (JS).

For the second tool, we utilized the SANRA scale for narrative reviews [20]—a validated six-item instrument for narrative reviews—to assess methodological transparency and narrative coherence. Two reviewers independently scored each article on the following: justification of the narrative format; clarity of aims; description of a literature search; quality of references; strength of scientific reasoning; and presentation of evidence. Items were scored 0–2, producing totals from 0 to 12. Divergent ratings were reconciled through discussion to ensure consistency.

For the third tool, the Joanna Briggs Institute guidance for scoping reviews [21]—which prioritizes transparent reporting of objectives, eligibility criteria, search strategies, and selection workflows but does not require formal bias appraisal—we confirmed complete adherence to JBI reporting standards. Both scoping reviews transparently documented their methodology; no domain-based risk-of-bias assessments were performed.

## 3. Results

### 3.1. Characteristics of Included Studies

A total of 39 reviews met our inclusion criteria: 33 identified through database searches and an additional 6 located via manual screening. This cumulative count (*n* = 39) excludes duplicates and ensures no overlap between reviews. These were published between 2021 and 2025 [1,2,3,4,5,6,7,8,22,23,24,25,26,27,28,29,30,31,32,33,34,35,36,37,38,39,40,41,42,43,44,45,46,47,48,49,50,51,52].

These reviews, comprising twenty systematic reviews, seventeen narrative reviews, and two scoping reviews, examined AI applications across the reproductive life course, from preconception and IVF laboratory workflows through prenatal ultrasound (automatic plane detection and biometry), obstetric risk prediction (preterm birth, preeclampsia, gestational diabetes), intrapartum forecasting (mode of delivery, induction outcomes), and neonatal/early child-development monitoring to clinical decision support systems, mHealth/chatbots for pregnancy and sexual/reproductive health, and ethical/governance frameworks. Data modalities ranged from imaging (ultrasound, histopathology, MRI) and electronic health records to physiological signals and wearable sensors; AI techniques included supervised machine-learning algorithms (logistic regression, support vector machines, random forests, gradient boosting), deep-learning architectures (CNNs, U-Nets, RNNs, transformers), and rule-based/NLP chatbots. (Supplementary Table S1).

### 3.2. Thematic Synthesis

#### 3.2.1. Overview

Thematic analysis of the 39 included reviews revealed four overarching themes that captured the breadth and depth of AI’s role in maternal–child health. First, we mapped AI innovations across the reproductive life course—from preconception and IVF laboratory workflows through pregnancy, fetal imaging and monitoring, to postpartum maternal wellness and neonatal/early child development. Second, we examined how AI is streamlining clinical operations and unlocking economic efficiencies in imaging workflows, laboratory automation, and remote monitoring. Third, we assessed the extent to which models have been validated, generalized, and applied equitably across diverse settings. Finally, we explored the technical, ethical, and regulatory considerations that will determine AI’s real-world impact, including integration with clinical systems, model interpretability, data governance, and accountability frameworks.

#### 3.2.2. Theme 1: Life-Cycle Stages of AI Application

Theme 1 synthesizes AI innovations across four pivotal life stages: reproductive and preconception care, pregnancy and fetal monitoring, postpartum maternal wellness, and neonatal and early child development.

##### Reproductive, Sexual, and Preconception Care

**Embryo and Gamete Assessment.** Convolutional neural network (CNN)-based platforms ERICA, STORK-A, KIDScore, and iDAScore achieve AUCs > 0.90 for euploidy and implantation prediction [51] and reduce manual cycle-cancellation risk factors by improving selection accuracy. Static-image CNNs classify blastocyst quality with ≈91% accuracy [46], and other time-lapse classifiers report viability-prediction AUCs up to 0.93 [42].

AI-powered CASA and smartphone semen analyzers deliver sperm motility and morphology classification to a precision of >98% [51]. Automated deep-learning models also achieve ≥89% accuracy on key sperm parameters [46]. The narrative review confirms widespread adoption of AI-driven Computer-Assisted Sperm Analysis (CASA) systems in clinical labs [35], underscoring gains in throughput and consistency.

**Gonadotropin Dosing and Protocol Optimization.** Wu et al., (2025) [8], a K-nearest neighbors model, personalized initial Follicle-Stimulating Hormone (FSH) dosing for each patient, yielding an average increase of 1.5 mature Metaphase II (MII) oocytes per cycle or conserving approximately 1 375 International Unit (IU) of FSH when lower doses sufficed, without reducing overall oocyte yield. Olawade et al., (2025) [49] described supervised machine-learning algorithms that integrate patient age, body mass index, antral follicle count, and anti-Müllerian hormone levels to optimize gonadotropin selection, dose adjustments, and trigger timing—thereby minimizing ovarian hyperstimulation risk while maximizing follicular recruitment. Bulletti et al., (2024) [44] introduced clinical decision-support tools embedding machine-learning–guided recommendations for initial FSH dosing, standardizing stimulation protocols and reducing inter-center variability. Finally, Kakkar et al., (2025) [35] outlined hybrid AI frameworks that combine clinical parameters with genetic profiling to individualize ovarian stimulation regimens—further enhancing mature oocyte yield and procedural safety.

**Digital Sexual and Reproductive Health (SRH).** Chawareb et al., (2025) [24] reported that the aDOT chatbot increased PrEP adherence by 91% and reduced condomless sex by up to 33% among MSM cohorts. Mills et al., (2023) [34], in their realist synthesis of chatbots, such as Sexpert and SnehAI, demonstrate that anonymous, nonjudgmental engagement fosters greater user disclosure and improved STI-prevention behaviors, although they do not provide exact metrics. Privacy and bias remain critical concerns: Chawareb et al., (2025) [24] stress the need for robust data protection (e.g., GDPR compliance) to safeguard sensitive sexual-health information and prevent misuse or re-identification. Empathy and trust are also at risk, as chatbots’ limited capacity for human-like compassion can produce insensitive or inaccurate responses that undermine user confidence. Finally, service integration poses a significant barrier: Mills et al., (2023) [34] emphasize that chatbots must be embedded within broader SRH service networks—linking users to human providers for issues beyond the chatbot’s scope—to achieve real-world uptake.

##### Pregnancy and Fetal Monitoring

**Advanced Imaging and Segmentation.** Generic deep-learning classifiers achieve a mean average precision of ≈ 0.955 and a recall of ≈ 0.931 for fetal-ultrasound plane detection [52]. Patel et al., (2024) [41] further validate CNNs at human-level sensitivity (≈95–96%, AUC 0.99) for identifying standard anatomical planes. Architectures, such as nnU-Net and 3D U-Net, deliver Dice coefficients > 0.90 for subcortical-structure delineation, matching or exceeding inter-observer variability [52]. Giaxi et al., (2025) [1] confirm these segmentation accuracies across external and cross-cohort validations. Frame-level inference times are under 15 ms—reducing manual scan workflows from minutes to real-time processing (≈13.6 ms/frame) [52]. Mapari et al., (2024) [40] replicate > 90% sensitivity for AI-augmented imaging in low-resource and rural settings.

**Anomaly Detection and Explainability.** ResNet classifiers detect hypoplastic left heart syndrome at 93.3% sensitivity and 100% specificity, while transformer-guided frameworks reach 96% sensitivity for chromosomal anomalies—both employing saliency-map heatmaps to highlight diagnostic features [52].

**Predictive Biometry and Risk Stratification.** Ensemble RNNs estimate gestational age with MAE < 1 week (R^2^ = 0.904) and CNNs predict fetal weight with RMSE < 190 g (MAPE ≈ 5.8%) [52]. Early-warning models—EHG-based random forests (AUC ≈ 0.99) and EHR-driven LSTM ensembles (AUC ≈ 0.827)—forecast preterm birth [39]. Machine-learning models for preeclampsia and gestational diabetes achieve AUCs of 0.89–0.99 in low-resource settings [40].

##### Postpartum and Maternal Wellness

**Mental Health Screening.** Du et al., (2023) [47] evaluate CDSS prototypes—such as an Android app for postpartum depression screening—demonstrating feasibility for early identification and referral. Mapari et al., (2024) [40] review predictive ML models and AI chatbots (e.g., Woebot, Wysa) for postpartum depression support, highlighting virtual mental-health interventions in low-resource settings.

**Hemorrhage and Morbidity Prediction.** Lin et al., (2024) [33] report that roughly 10 percent of AI-augmented CDSS studies address postpartum care—specifically hemorrhage risk assessment—but note a dearth of external validation and performance metrics. Giaxi et al., (2025) [1] and Patel et al., (2024) [41] similarly emphasize AI’s potential to forecast severe maternal events, while calling for standardized validation in real-world workflows.

**Remote Monitoring and Telehealth.** Ahmad et al., (2022) [25] demonstrate wearable sensor networks for continuous maternal vital-sign monitoring (blood pressure, heart rate) with real-time clinician alerts, enabling home-based surveillance. Du et al., (2023) [47] describe mobile CDSS apps (e.g., OG-Miner, Sinedie) and telehealth platforms for ongoing postpartum care and mental-health support, though quantitative evidence on reductions in in-person visits remains unexplored.

##### Neonatal and Early Child Development

**NICU Decision Support.** The HeRO score’s AI-driven heart-rate characteristic analysis reduced all-cause neonatal mortality by ~20 percent in a multicenter RCT [32]. Published sepsis-prediction models report AUCs > 0.80 for NEC and ROP detection, though individual accuracies vary by condition and dataset.

**Infant Disease Detection and Prediction.** Deep neural networks, random forests, and support vector machines achieve 63–94 percent accuracy for autism spectrum disorder screening via computer-vision analysis of facial and motion data [45]. Smartphone-based bilirubin screening [36] and national-survey ML models for malnutrition classification offer promising avenues but lack standardized performance reporting.

**Developmental Monitoring.** Computer-vision and wearable-sensor tools track motor, language, and social development in cross-sectional studies [45]—but few systems have undergone longitudinal validation or broad real-world deployment.

#### 3.2.3. Theme 2. Operational Efficiency and Economic Outcomes

Across three key subthemes—imaging workflow acceleration, laboratory automation in assisted reproduction, and resource allocation with remote monitoring—AI is revolutionizing clinical operations and unlocking new economic efficiencies.

##### Imaging Workflow Acceleration

State-of-the-art deep-learning pipelines now automate fetal biometry end-to-end: U-Net-based segmentation delivers head-circumference measurements with a mean absolute error of ≈2 mm and femur-length measurements within ≈0.5 mm, processing each ultrasound frame in ≈13.6 ms—versus the several minutes required for manual caliper-based protocols [52]. In parallel, blind-sweep ultrasound algorithms estimate gestational age with a mean absolute error of ≈2.55 days, matching specialist-led 2D biometry and enabling accurate, high-throughput assessment even in low-resource settings [37].

##### Laboratory Automation in Assisted Reproduction

**Personalized FSH Dosing:** A K-nearest neighbors’ decision-support model tailors initial FSH regimens—yielding an average gain of 1.5 mature (MII) oocytes per cycle or conserving approximately 1 375 IU of FSH without reducing total yield [8].

**Embryo Selection:** CNN-based platforms (ERICA, STORK-A, KIDScore, iDAScore) achieve AUCs > 0.90 for implantation and euploidy prediction, translating into higher cycle success rates [42,51].

**Semen Analysis:** AI-powered CASA and smartphone-based analyzers classify sperm motility and morphology with > 98% precision—standardizing assessments and reducing manual workload [51], and automated semen-analysis systems achieve ≥ 89% accuracy in concentration and motility classification [46].

##### Resource Allocation and Remote Monitoring

Machine learning and connected technologies are streamlining resource use and extending care beyond the clinic walls.

**Intelligent Lab Scheduling.** AI-driven workflow optimizers in IVF laboratories forecast peak activity, allocate staff and equipment dynamically, and reschedule tasks in response to disruptions—minimizing wait times and idle capacity [49].

**Continuous Vital-Sign Surveillance.** Wearable platforms, such as the VO7 blood-pressure and heart rate monitor and the Invu acoustic-electrical sensor system, deliver vital-sign measurements with correlation coefficients of r ≈ 0.92–0.97 versus clinical standards, enable remote clinician review of data, and have been shown to reduce maternal mortality by 7% and preeclampsia rates by 11% in pilot evaluations [25].

**AI-Enhanced Telehealth.** AI-driven telehealth platforms and chatbots are redefining maternal care by delivering seamless, personalized support throughout pregnancy and the postpartum period. Interactive systems combine real-time symptom triage with tailored educational modules and virtual counseling—empowering women to receive expert guidance on warning signs, medication management, and emotional well-being anytime, anywhere [40]. AI-powered conversational agents reinforce care engagement through customized voice/text reminders, structured symptom logging, and empathetic psychosocial support—driving higher adherence to care plans and improved mental-health outcomes among new mothers [22]. Early demonstrations of lab automation and remote monitoring suggest substantial efficiencies—freeing clinician time, lowering per-cycle overhead, and decreasing patient travel and clinic load—though formal cost-effectiveness studies are still needed to quantify system-wide savings.

#### 3.2.4. Validation, Generalizability, and Equity

Across the three critical subthemes—limited external validation, domain adaptation and transferability, and geographic distribution and equity—AI in maternal–fetal care confronts both methodological and ethical hurdles on the path to real-world impact.

Most AI studies in maternal–fetal care remain confined to retrospective, single-center cohorts; external validation is rare, with only about 16% of clinical decision-support reviews reporting independent testing on held-out datasets [33]. Meta-analyses of gestational-age estimation models reveal very high between-study heterogeneity (I^2^ ≈ 97.9%), underscoring divergent methods and the need for prospective, multicenter trials [37]. Notable examples of prospective validation include the HeRO sepsis RCT, which demonstrated a ≈20% mortality reduction [32] and ongoing randomized evaluations of algorithm-guided FSH dosing in IVF [8].

##### Domain Adaptation and Transferability

Transfer-learning experiments reveal significant domain shift: models trained on high-resource ultrasound datasets drop recall to approximately 0.80 when applied in African cohorts (Tadepalli et al. [52]). More broadly, external or cross-cohort validations remain rare—most peripartum and neonatal AI tools rely solely on retrospective, single-center data [39].

##### Geographic Distribution and Equity

AI research in obstetrics and neonatology is heavily concentrated in Asia, Europe, and North America, whereas Sub-Saharan Africa is represented by just two centers (Morocco, Kenya) among 43 studies [50]. Equity analysis, such as demographic subgroup reporting and formal fairness audits, is rarely conducted, risking the perpetuation of algorithmic bias and widening health disparities [1].

#### 3.2.5. Implementation and Ethical–Regulatory Considerations

This theme examines the real-world deployment and governance of AI in maternal–fetal care by focusing on seamless technical integration with laboratory information management systems LIMS and EHRs, transparent, interpretable models to secure clinician trust, and rigorous regulatory, privacy, and ethical frameworks.

##### Technical Integration and Workflow Fit

Most AI solutions in maternal–fetal care remain at proof-of-concept, with few demonstrating true integration into clinical IT ecosystems. Hew et al., (2024) [42] report that AI-based IVF laboratory tools often lack seamless links to LIMS, impeding workflow fit. Patel et al., (2024) [41] highlight similar gaps in EHR integration for obstetric AI prototypes, and Giaxi et al., (2025) [1] note that only a handful of studies describe real-world software deployments within hospital infrastructures.

##### Regulatory Frameworks, Privacy, and Ethics

Data-Protection Compliance. Reviews of genomic and IVF AI applications call for robust privacy and consent frameworks—Bou Nassif et al., (2022) [48] highlight genetic/imaging data-privacy needs, and Ahmad et al., (2022) [25] emphasize security and regulation gaps—but neither quantifies GDPR nor HIPAA adherence, and explicit references to these regulations are sparse.

**Device-Approval Pathways.** Narrative and systematic reviews of prenatal- and neonatal-care AI [32,41] focus on proof-of-concept models and do not delineate clear medical-device-approval routes for ultrasound, CTG, or NICU monitoring tools.

**Ethical Frameworks vs. Practice.** Chng et al., (2025) [26] propose the PEARL-AI framework—detailing principles for child-centered AI governance (consent, fairness auditing, accountability)—yet real-world uptake of these concrete consent processes, bias-mitigation audits, and accountability mechanisms remains very limited (Table 3).

### 3.3. Quality Assessment Results

In our AMSTAR 2 evaluation of 39 reviews (20 systematic, 2 scoping, 17 narrative), critical deficiencies again dominated. Only 4/39 (10.3%) preregistered a protocol (Item 2) [2,31,37,44]. While scoping and narrative reviews are not always preregistered per JBI and SANRA guidance, the overall paucity of protocol registration may introduce selective reporting and other biases.

Dual independent screening (Item 5) was reported in 11/39 (28.2%) reviews [1,2,3,4,5,31,33,35,37,38,45]. Formal appraisal of primary-study risk of bias (Item 9) appeared in only 6/39 (15.4%) reviews [2,3,5,27,37,45] and none investigated publication bias (Item 15), leaving every synthesis vulnerable to selective reporting.

Consequently, 33 reviews (84.6%) were deemed critically low confidence, 3 (7.7%) moderate confidence, and only 3 (7.7%) [2,31,44] achieved high confidence by preregistering protocols, deploying dual-reviewer methods, and applying formal risk bias tools.

### 3.4. Risk of Bias Across Review Types

When appraised with ROBIS (Supplementary Table S3A), the 20 systematic reviews yielded the following overall judgments: two [2,37] demonstrated low risk; one [3] was moderate risk; two [27,45] were unclear; and the remaining fifteen (75%) were high risk.

Domain-level performance was as follows:

- Domain 1 (Eligibility Criteria): 16/20 (80%) clearly pre-specified PICOS elements; 3/20 (15%) were high risk; 1/20 (5%) was unclear.
- Domain 2 (Identification and Selection), 19 of 20 systematic reviews (95%) reported comprehensive, multi-database searches and provided PRISMA flow diagrams. The one exception offered only a partial search description, which we noted under ‘high risk’ for Domain 2
- Domain 3 (Data Collection and Appraisal): Only 6/20 (30%) performed a structured critical appraisal of included studies; 14/20 (70%) did not.
- Domain 4 (Synthesis and Findings): 4/20 (20%) conducted sensitivity or heterogeneity analyses; 16/20 (80%) omitted these procedures.

Among the 17 narrative reviews evaluated via SANRA (Supplementary Table S3B), scores ranged from 8 to 12 out of 12 (mean ≈ 9.4). All 17 reviews

- Made a compelling case for their article type (Item 1).
- Stated explicit aims (Item 2).
- Employed coherent scientific reasoning (Item 5).
- Organized and presented evidence effectively (Item 6).

However, none (0/17) described their literature-search methods in sufficient detail (Item 3), undermining reproducibility. These robust SANRA ratings stand in stark contrast to the universal “critically low confidence” assigned by AMSTAR 2, which penalizes absent protocols, lack of formal bias appraisal, and failure to assess publication bias—dimensions beyond SANRA’s remit.

The two scoping reviews adhered to JBI guidance (Supplementary Materials Table S3C), transparently reporting scope, eligibility criteria, search strategy, and selection methods but—consistent with JBI recommendations—did not conduct formal risk-of-bias assessments. Under AMSTAR 2, this omission resulted in both being rated “critically low confidence,” illustrating how divergent appraisal tools can produce complementary yet occasionally discordant verdicts.

## 4. Discussion

This scoping review of reviews integrated findings from thirty-nine reviews—spanning systematic, scoping, and narrative methodologies—to chart the application of AI and ML across reproductive, maternal, fetal, neonatal, and early child-development care. Four overarching insights emerged from this synthesis. These insights are directly aligned with our objectives. Only ten percent of the reviews preregistered protocols, <30 percent used dual-reviewer screening, and <5 percent assessed publication bias—limiting the certainty of aggregate conclusions and reinforcing the need for rigorous future studies.

AI models exhibit exceptional retrospective accuracy across the perinatal continuum. CNNs designed for embryo viability prediction report AUC values exceeding 0.90, while advanced segmentation frameworks like U-Net and transformer architectures achieve Dice coefficients above 0.90 for fetal ultrasound analysis—often in under 15 milliseconds per frame. Neonatal sepsis-prediction models similarly demonstrate AUCs at approximately 0.82 for 24 h advance forecasting [32].

Automated AI pipelines promise to streamline time-intensive workflows. Real-time ultrasound segmentation can feed images directly into diagnostic dashboards, AI-driven embryo scoring has been associated with an average increase of 1.5 viable oocytes per in vitro fertilization cycle, and remote monitoring platforms correlate maternal vital signs with clinical measurements at r ≈ 0.92–0.97 [8,22]. Despite these encouraging pilot outcomes, rigorous health–economic evaluations—such as cost-effectiveness or budget-impact studies—are notably scarce. Despite reports of workflow savings, no formal cost-effectiveness analyses have been published for IVF laboratory automation (e.g., ERICA, STORK-A) or neonatal sepsis-prediction tools such as HeRO. Pilot surveys report >80 percent satisfaction with AI-guided embryo scoring dashboards [53]. A mobile CDSS for postpartum depression reduced PHQ-9 scores by four points on average [47].

External validation remains an unresolved challenge: when decision-support models developed in well-resourced settings are applied to low-resource cohorts, performance can decline sharply—from recall values of 0.95 down to 0.80 [33].

Equity-focused validation should be incorporated by evaluating model performance across socio-demographic subgroups and varied resource settings to identify and correct bias. Robust governance mechanisms are needed, including preregistered protocols, transparent performance reporting, and routine fairness audits aligned with TRIPOD-AI and CONSORT-AI standards. To translate technical gains into clinical impact, practical integration must be pursued through seamless embedding of AI outputs into EHR dashboards, clinician education initiatives, and patient-centered monitoring tools, thereby orienting AI development toward enhanced perinatal outcomes rather than accuracy alone. AI systems must shift from opaque “black boxes” to interpretable models (e.g., via SHAP, LIME, saliency mapping). This consolidation underscores that the lack of transparency undermines clinician trust, impedes regulatory approval, and jeopardizes equitable adoption across settings.

Seamless integration with EHRs or LIMS is reported in fewer than 10 percent of studies. Regulatory pathways for adaptive, continuously learning algorithms remain ill-defined, complicating the approval process for evolving AI tools [54]. Inconsistent data governance policies, consent frameworks, and liability models across jurisdictions further impede clinicians and developers from translating AI prototypes into everyday practice.

The transformative potential of AI is best realized when interventions are orchestrated across the full reproductive–perinatal–neonatal continuum rather than deployed in isolation.

In the preconception phase, CNN platforms, such as ERICA and iDAScore, achieve implantation-prediction AUCs above 0.90, guiding embryo selection and personalizing ovarian stimulation protocols [42]. Complementary fertility applications and virtual assistants leverage patient data to tailor counseling and optimize treatment regimens. However, few longitudinal studies have examined the downstream impact of these AI-driven preconception decisions on perinatal and neonatal outcomes.

Machine-learning models (e.g., K-nearest neighbors) personalized FSH dosing, yielding +1.5 MII oocytes per cycle and ~1 375 IU FSH savings without compromising yield [8].

During pregnancy, automated ultrasound algorithms employ U-Net or transformer models to reliably segment fetal structures for anomaly detection, with inference times compatible with real-time clinical use [52]. Machine-learning models predicting conditions such as pre-eclampsia and gestational diabetes achieve AUCs of up to 0.93 [55]. Yet, randomized trials confirming that AI-guided screening interventions reduce morbidity or mortality remain exceptionally rare.

Validated AI tools for continuous labor monitoring—such as automated recognition of contraction patterns or cardiotocography interpretation—are notably underdeveloped. Few prototypes have moved beyond small cohort validations, leaving critical intrapartum decision-support needs, like timely identification of fetal distress, unaddressed.

In neonatal intensive care, the HeRO score stands as a prominent success, reducing mortality by approximately 20 percent in a multicenter randomized trial through early sepsis detection [32]. Nevertheless, most neonatal AI solutions remain confined to pilot studies and are not yet integrated into comprehensive ICU workflows. Prospective evaluations are required to confirm their safety and efficacy in diverse clinical settings.

Rather than asking which algorithm achieves the highest standalone accuracy, stakeholders must consider how to link AI tools into sequential care pathways. For example, a preterm birth risk model could trigger prophylactic corticosteroid administration, followed by AI-guided labor monitoring and postnatal sepsis surveillance, creating a cascade of interventions that collectively amplify patient benefit. Designing and testing such integrated pipelines should become a priority for future research.

Achieving population-level health improvements through AI requires embedding these technologies within resilient health systems and safeguarding equitable access.

To prevent AI from deepening disparities, training datasets must encompass the full spectrum of urban and rural, resource-rich and resource-constrained, and ethnically diverse populations. Tools, such as the HEAL audit framework, can systematically evaluate subgroup performance, guiding iterative model refinements to ensure that benefits accrue uniformly across all communities [56]. Regulatory agencies can reinforce these efforts by mandating fairness assessments for all AI applications prior to approval.

In many low- and middle-income countries (LMICs), intermittent power supplies, limited broadband connectivity, and reliance on paper-based record-keeping pose formidable challenges. Establishing digital foundations—robust EHR systems, reliable telecommunication networks, and consistent electricity—must accompany any AI deployment strategy. Equally critical is workforce development: clinicians, data managers, and IT personnel require comprehensive training in AI literacy, data governance, and system maintenance to ensure sustainable adoption.

Maternal and child health challenges transcend national borders. Federated learning consortia, coordinated by international bodies such as the World Health Organization, enable the development of algorithms on distributed datasets while preserving patient privacy. Harmonizing reporting standards through guidelines like TRIPOD-AI and CONSORT-AI further enhances inter-study comparability and accelerates model generalizability across diverse healthcare environments.

Even the most rigorously validated AI models confront multifaceted barriers when transitioning from research to routine care.

Perinatal data often reside in fragmented paper records, isolated digital silos, and non-standardized registries, preventing the creation of comprehensive, longitudinal datasets necessary for robust model training. In addition, inconsistent coding practices and measurement noise—such as ultrasound artifacts—degrade algorithm performance in real-world applications. Addressing these shortcomings demands the development of integrated mother–child registries with standardized data elements and the deployment of federated data networks that allow collaborative analytics without centralizing sensitive information.

Adaptive AI algorithms that continuously learn from new data disrupt traditional medical-device classification, which is predicated on static technologies. Regulatory bodies and industry stakeholders must co-develop lifecycle-oriented frameworks that specify requirements for prospective validation trials, audit logs, and ongoing safety monitoring. Early engagement between developers and regulators can clarify expectations and expedite approval processes for evolving AI tools.

Clinicians operating under time pressures are unlikely to adopt AI tools that demand manual data entry or disrupt established workflows. Employing human-centered design principles—co-developing interfaces with end users, piloting prototypes in situ, and iterating based on feedback—can facilitate smoother integration. Establishing local “AI champions” and offering structured training workshops helps build trust and competency among frontline staff.

Algorithmic decision-support in sensitive reproductive contexts risks being perceived as impersonal or intrusive. Moreover, the confidentiality of reproductive and genomic data heightens privacy concerns. Early engagement with patient advisory boards and community focus groups can surface local values, ethical expectations, and cultural reservations, ensuring that AI deployments are both acceptable and respectful of participants’ rights.

The costs of hardware procurement, software licensing, and ongoing maintenance represent significant financial commitments. Without a clear analysis of return on investment, health systems may hesitate to allocate the necessary resources. Innovative financing models—such as public–private partnerships, open-source platforms, and value-based reimbursement schemes—offer potential pathways to distribute costs more equitably and incentivize long-term sustainability.

In low-resource settings, AI can be scaled affordably and effectively by leveraging ubiquitous hardware and offline-capable algorithms: for example, a smartphone-based “blind-sweep” ultrasound paired with a deep-learning model achieved a gestational-age MAE of 2.55 days without requiring trained sonographers in rural African clinics [37]; a simple smartphone image analysis of palpebral conjunctiva predicted maternal hemoglobin with r≈0.90, enabling community health workers in Indian villages to screen for anemia without laboratory infrastructure [36]; a mobile app using sclera-chromaticity analysis detected neonatal jaundice with 100% sensitivity to trigger timely phototherapy referrals where bilirubin assays are scarce [36]; and low-power wearable IoT sensor networks with on-device analytics provided continuous blood-pressure and heart-rate monitoring in rural Ghana, cutting clinic visits by 30% and improving preeclampsia management [25]. To ensure responsible scale-up in low- and middle-income countries, obstetric AI initiatives should mirror successful models from other clinical domains. Non-real-time, EHR-driven surveillance systems for sepsis and device-associated infections could be repurposed for maternal sepsis monitoring, offering cost-effective, scalable alerts in settings lacking continuous specialist oversight [57]. Moreover, open-data consortium strategies and bias-audit practices championed in vaccine R&D reviews provide a roadmap for developing shared, multi-center repositories that bolster model generalizability and equity in obstetric AI applications [58].

## 5. Methodological Reflexivity

Although formal quality assessment and risk-of-bias appraisal are not mandatory under PRISMA-ScR, our scoping review of existing reviews includes a methodological quality evaluation for enhanced transparency and contextual insight. This approach aligns with emerging best practices when scoping reviews aim to not only map evidence but also comment on its reliability, thus informing interpretations of findings and identifying methodological gaps.

## 6. Policy and Implementation Implications

Translating AI’s extraordinary technical capabilities into real-world gains for maternal and neonatal health demands not only vision but rigor. Regulatory authorities must insist that every AI tool undergoes a preregistered evaluation protocol, dual independent screening of evidence, and formal risk-of-bias appraisal—remediating the systemic deficiencies that undermine confidence in existing syntheses. Approval should be conditional on demonstrable subgroup fairness audits and transparency of decision logic (e.g., open source explainability reports), thereby ensuring that algorithms validated predominantly in single-center, retrospective cohorts generalize equitably across diverse populations.

At the same time, governments and funders must invest in foundational infrastructure—robust EHR integration, reliable broadband, and uninterrupted power—especially in under-resourced regions, to prevent perpetuating the “data silo” and “infrastructure gap” issues highlighted by our quality appraisals. Existing medical-device frameworks should be overhauled to require external, prospective multicenter validation and ongoing post-market surveillance, closing the loop on adaptive, continuously learning AI systems whose performance drift has been left unchecked. Multi-stakeholder task forces—bringing together clinicians, methodologists, ethicists, patient advocates, and regulators—should oversee the development of standardized reporting checklists (aligned with PRISMA-P, PRISMA-ScR, TRIPOD-AI, and CONSORT-AI), mandate the creation of anonymized, federated maternal–child data repositories, and deploy real-time performance dashboards with automated bias-detection pipelines. Only through such coordinated, methodologically rigorous governance can AI advance patient autonomy, safeguard data privacy, and drive equitable improvements in perinatal outcomes.

## 7. Strengths and Limitations

By synthesizing thirty-nine systematic, scoping, and narrative reviews and applying four complementary appraisal instruments (AMSTAR 2, ROBIS, SANRA, JBI), this study offers the most comprehensive, critically aware panorama yet of AI in perinatal care. However, the alarmingly low methodological quality of the underlying evidence—84.6% of reviews rated critically low confidence, pervasive absence of protocol preregistration (10.3%), scant duplicate review processes, and universal neglect of publication-bias assessments—reveals a profound transparency gap. These deficits not only temper our conclusions but also signal an urgent research imperative: future evidence syntheses must preregister protocols, enforce dual-reviewer workflows, conduct formal bias and overlap analyses of primary studies, and adhere rigorously to reporting guidelines. Only by elevating the methodological foundations of AI research can we build the reliable evidence base required to transform perinatal care safely, equitably, and on scale.

## 8. Future Research, and Integration

**Phase I: Consolidation and Validation** calls for the establishment of international consortia to develop federated, de-identified maternal–newborn datasets reflecting diverse demographics; mandatory protocol preregistration and dual-reviewer methodologies to enhance transparency; and the initiation of adaptive platform trials and stepped-wedge studies targeting high-impact applications, such as pre-eclampsia prediction and neonatal sepsis alerts.

**Phase II: Pilot Implementation and Integration** involves deploying validated AI pipelines in early-adopter hospitals with direct integration into EHR and LIMS modules, supported by implementation-science evaluations that assess workflow redesign, clinician acceptance, and patient experience. Continuous monitoring dashboards should track model performance, equity metrics, and safety signals in real time.

**Phase III: Scale-Up and Governance** requires the development of interoperable AI platforms featuring embedded explainability tools and automated compliance checks, harmonization of global regulatory frameworks—guided by WHO digital health guidelines—to facilitate multi-jurisdictional approval and secure data sharing, and the creation of targeted incentives (grant programs, reimbursement schemes, fast-track review processes) to promote equity-focused AI solutions in high-burden, underserved regions.

## 9. Conclusions

This scoping review of reviews offers a critical, panoramic assessment of AI’s present and potential impact on reproductive, maternal, fetal, neonatal, and early child-development care. While retrospective studies consistently demonstrate remarkable predictive accuracy and operational efficiencies, the translation of AI into routine clinical practice remains fragmented by validation deficits, equity gaps, infrastructural constraints, and regulatory uncertainties. To fulfill AI’s paradigm-shifting promise—safer pregnancies, optimized resource allocation, and healthier neonates—stakeholders must pursue an integrated, equity-centered, and systems-oriented strategy. Implementing a forward-looking roadmap that combines rigorous validation, inclusive governance, strategic infrastructure investment, and robust ethical frameworks will empower the global health community to accelerate the safe, equitable, and effective integration of AI across the entire perinatal life course.

## Figures and Tables

**Table 1:** SPIDER Framework for Eligibility Criteria.

Component	Definition
Sample	Systematic reviews (with or without meta-analysis), scoping reviews, and narrative reviews of AI/ML interventions in human reproductive, prenatal, postpartum, neonatal, or early-child-development care.
Phenomenon of Interest	AI-driven algorithms (machine learning, deep learning, neural networks, ensemble methods) applied to diagnosis, prediction, or workflow optimization.
Design	Evidence syntheses published in peer-reviewed journals; primary studies were excluded.
Evaluation	Quantitative: AUC; sensitivity; specificity; Dice coefficient; MAE; inference/processing time; workflow-time savings; cost-effectiveness. Qualitative: Implementation barriers/facilitators; equity/fairness analyses; user acceptability; ethical/regulatory themes.
Research type	Reviews using quantitative, qualitative, or mixed-method synthesis that report at least one of the above quantitative metrics or qualitative themes.

**Table 2:** MEDLINE Search Strategy.

Concept	MeSH Terms	Free-Text Terms
Artificial intelligence	“Artificial Intelligence” [Mesh]	“Machine learning”, “deep learning”, “neural network”
Perinatal health	“Reproductive Health” [Mesh], “Pregnancy” [Mesh], “Infant, Newborn” [Mesh]	“IVF”, “embryo”, “fetal monitoring”, “neonatal intensive care”, “NICU”
Study type	-	review [pt], “systematic review”, “scoping review”, “narrative review”, “meta-analysis”,

**Table 3:** Plain-Language Summary of Findings.

Findings	Plain Language
**1. Reproductive and Preconception Care**	Computer tools now examine embryos and sperm to pick the healthiest ones. These tools are correct over 90% of the time, helping clinics reduce canceled cycles and improving pregnancy chances.
	Other models adjust hormone doses for each patient so that she produces more mature eggs without extra medication.
	Chatbots help people stick to HIV prevention steps and learn about sexual health—but they still need better protection of private information and must connect with real clinics.
**2. Pregnancy and Fetal Monitoring**	New image-analysis software can draw an outline around the fetus on ultrasound frames in about 14 ms—almost instantly—so measurements are more consistent and take far less time than manual methods.
	Some tools spot heart or brain abnormalities with over 93% accuracy, similar to specialist readings, and even highlight the suspicious area on the image.
	Models can predict how far along the pregnancy is (within one week) and estimate fetal weight (within about 190 g), helping providers identify growth issues earlier.
**3. Postpartum and Maternal Wellness**	Apps and chatbots can screen for postpartum depression, alerting healthcare workers when a new mother needs extra support.
	Some AI tools try to predict risks like heavy bleeding or serious complications, but most have not yet been tested outside of small research groups.
	Wearable devices allow mothers to monitor blood pressure and heart rate at home, sending real-time alerts to clinicians; early pilots show fewer serious problems with these systems, but more research is needed.
**4. Neonatal and Early Child Development**	In neonatal ICUs, the HeRO monitor (which analyzes infant heart-rate patterns) reduced death rates in very low-birth-weight babies by about 20% in a major trial.
	Other models flag infections or eye problems (like retinopathy of prematurity) with approximately 80% accuracy before symptoms appear.
	Computer vision and wearable sensors can track a baby’s movements and milestones (such as crawling or babbling), but most of these tools have not yet been tested over long periods or in many hospitals.
**5. Operational Efficiency**.	Automated ultrasound measurement means a sonographer spends seconds—rather than minutes—obtaining fetal size data, freeing up appointments.
	In fertility labs, AI schedules tasks (like when to incubate embryos) so staff spend less time on paperwork and more time on patient care.
	Remote-monitoring platforms for pregnant women (using wearables and apps) have been linked to 7–11% fewer maternal deaths and preeclampsia cases, because clinicians can intervene earlier when warning signs appear.
**6. Validation, Generalizability and Equity**	Most AI tools have been tested only in a single hospital or research center, so they may not work as well in other regions or different patient groups.
	When the same algorithm is moved from a well-resourced hospital to a rural clinic, its accuracy often falls (for example, from 95% down to 80%).
	Very few studies come from Sub-Saharan Africa or other low-resource settings—raising concerns that certain populations could be left behind or see less accurate results.
**7. Implementation and Governance**	Although many promising AI tools exist on paper, hardly any are fully hooked up to hospital record systems—so clinicians must still enter data manually or use separate software.
	There are rules on data privacy and ethical use (for example, avoiding biased outcomes), but these guidelines are rarely enforced, delaying widespread use.
	To bring AI into everyday care, hospitals need better IT systems (reliable EHRs and lab networks), clear rules on how to approve changing algorithms, and training for clinicians so they trust and understand these tools.

## Data Availability

The authors confirm that the data supporting the findings of this study are available within the article and its Supplementary Materials. The codes used in the analysis of this study will be made available from the corresponding author upon reasonable request.

## Supplementary Materials

The following supporting information can be downloaded at: https://www.mdpi.com/article/10.3390/nursrep15080281/s1, Table S1: Characteristics of included studies. Table S2: AMSTAR 2(A Measurement Tool to Assess Systematic Reviews 2). Table S3A: ROBIS. Table S3B: SANARA. Table S3C: Scoping JBI. References [1,2,3,4,5,6,7,8,22,23,24,25,26,27,28,29,30,31,32,33,34,35,36,37,38,39,40,41,42,43,44,45,46,47,48,49,50,51,52] are cited in the main text as included reviews and are additionally cited in the Supplementary Materials to provide further methodological and descriptive characteristics.

## Public Involvement Statement

No public involvement in any aspect of this research.

## Guidelines and Standards Statement

This manuscript was drafted against PRISMA for scoping review research.

## Glossary (Plain Language Definitions)

Artificial Intelligence (AI)	Computer programs designed to mimic human thinking and decision-making, such as recognizing patterns in medical images or predicting patient risks.
Machine Learning (ML)	A subset of AI where computers “learn” from large amounts of data (like past patient records) to make predictions or recommendations without being explicitly programmed for every scenario.
Convolutional Neural Network (CNN)	A type of computer model especially good at analyzing medical images—think of it as software that “looks” at ultrasound or embryo pictures and highlights important features.
Recurrent Neural Network (RNN)/Long Short-Term Memory (LSTM)	Computer models that work well with data that comes in a sequence over time (for example, heart-rate changes during labour). They help predict events (such as when labor might start).
Transformer Model (e.g., ResNet, U-Net)	Advanced computer systems that process information (like ultrasound frames) very quickly and can point out abnormalities—imagine a tool that instantly draws outlines around the fetus in an ultrasound image.
Area Under the Curve (AUC)	A score (from 0 to 1) that tells us how well a test or model can tell “sick” versus “healthy.” An AUC of 0.80 means the tool is correct 80% of the time at distinguishing disease.
Dice Coefficient (also called Dice Score)	A number (0 to 1) that measures how closely a computer’s outline of an organ (for example, the fetal head) matches an expert’s outline. A Dice score of 0.90 means 90% overlap—very close agreement.
Sensitivity	The ability of a test or model to correctly identify those who have the condition. If sensitivity is 93%, it catches 93 out of 100 true cases.
Mean Absolute Error (MAE)	The average amount by which a prediction (such as fetal age in weeks) differs from the true value. If MAE is less than 1 week, the prediction is usually within one week of the actual age.
Clinical Decision Support System (CDSS)	A software tool that provides healthcare workers with personalized recommendations—such as reminding a midwife when to screen for postpartum depression or alerting a nurse to possible neonatal sepsis.
Embryo Selection Platform (e.g., ERICA, STORK-A, KIDScore, iDAScore)	Computer tools used in fertility clinics to choose the healthiest embryo(s) by analyzing images and data, aiming for higher chances of successful pregnancy.
Clinical Practice Guidelines (e.g., TRIPOD-AI, CONSORT-AI)	Checklists and rules for how to report and evaluate AI tools so that doctors and nurses can trust they work safely and fairly.
Electronic Health Record (EHR)/Laboratory Information Management System (LIMS)	Digital systems that store patient information and lab data. When AI is “integrated” with EHRs/LIMS, it means these tools can automatically pull and analyze patient data without extra manual steps.
Federated Learning	A way for hospitals to train AI tools on their own patient data without sharing the actual data with each other—only the “lessons learned” go back to a central system. This protects privacy while improving model accuracy across different regions.

## Abbreviations

AUC	Area Under the (Receiver Operating Characteristic) Curve
AMSTAR 2	A Measurement Tool to Assess Systematic Reviews 2
CDSS	Clinical Decision Support System
CNN	Convolutional Neural Network
CONSORT-AI	Consolidated Standards of Reporting Trials—AI extension
EHR	Electronic Health Record(s)
EHG	Electrohysterography
GDPR	General Data Protection Regulation
HBPA—HIPAA	Health Insurance Portability and Accountability Act
ICU	Intensive Care Unit
IVF	In Vitro Fertilization
IU	International Unit
JBI	Joanna Briggs Institute
KNN	K-Nearest Neighbors
LIMS	Laboratory Information Management System
LSTM	Long Short-Term Memory
MAE	Mean Absolute Error
MAPE	Mean Absolute Percentage Error
MII	Metaphase II Oocyte
mHealth	Mobile Health
ML	Machine Learning
NEC	Necrotizing Enterocolitis
NICU	Neonatal Intensive Care Unit
NR-PRISMA	Preferred Reporting Items for Systematic Reviews and Meta-Analyses (PRISMA 2020)
PHQ-9	Patient Health Questionnaire-9
PICU	Pediatric Intensive Care Unit
PRISMA	Preferred Reporting Items for Systematic Reviews and Meta-Analyses
PROSPERO	International prospective register of systematic reviews (for protocol registration)
PSROC	Patient-Reported Outcomes and Clinical (where context applies—often in economic evaluations)
RNN	Recurrent Neural Network
ROBIS	Risk Of Bias In Systematic Reviews
ROP	Retinopathy of Prematurity
RMSE	Root Mean Square Error
RCT	Randomized Controlled Trial
SANRA	Scale for the Assessment of Narrative Review Articles
SPIDER	Sample, Phenomenon of Interest, Design, Evaluation, Research type
TRIPOD-AI	Transparent Reporting of a multivariable prediction model for Individual Prognosis Or Diagnosis—AI extension
U-Net	Convolutional Network for Biomedical Image Segmentation (“U-Net” architecture)
