# Supplementary material for: Integrating Artificial Intelligence into Perinatal Care Pathways: A Scoping Review of Reviews of Applications, Outcomes, and Equity
Source: Nurs Rep. 2025 Jul 31;15(8):281. doi: 10.3390/nursrep15080281 (PMC12388636; doi:10.3390/nursrep15080281)
Supplement: Supplementary file 1 [file nursrep-15-00281-s001.zip › nursrep-3706470-supplementary.pdf]

Supplementary Table S1: Characteristics of included studies

Citation (Author et al., Year)	Design & Scope	AI Application(s)	Model Types & Key Performance	Clinical & Economic Outcomes	Validation & Generalizability	Geographic Setting & Equity	Implementation Barriers	Ethical / Regulatory Issues
Yaseen & Rather, 2024 (Yaseen & Rather, 2024)	Theoretical review of AI's role across conception to delivery	ML, NLP, ANN, computer	Overview of ANN, SVM, RF, boosting, deep learning high AUCs (~0.98)	Improved diagnostic accuracy, workflow efficiency, and real-time monitoring potential; no formal economic impact assessed	Predominantly internal validations; few external/multicenter studies; heterogeneous, single-center data limit generalizability	Research largely from China with sporadic global contributions; equity, demographic, and resource-setting impact largely unreported	Data quality/heterogeneity, interoperability issues, lack of standardized protocols, explainability gaps, workflow integration and regulatory complexity	Underaddressed patient privacy/consent, bias mitigation, adherence to AI reporting guidelines (SPIRIT-AI, CONSORT-AI), and need for robust regulatory oversight
Xiao et al., 2023 (S. Xiao et al., 2023)	Narrative review of AI in prenatal fetal ultrasound, focusing on automatic standard-plane detection, biometric measurement, and disease diagnosis;	Standard-plane detection; biometric parameter measurement (HC, AC, NT); disease diagnosis (fetal lung maturity, intracranial anomalies, GA estimation, CHD)	Predominantly deep learning (CNN, U-Net, RNN, transformers); reported accuracies up to ~99% (plane detection), DSC > 97% (biometry), AUC ≈ 0.99 (diagnosis)	Integration of AI tools (e.g., commercial "SmartPlanes," SonoNT, FINE) has improved workflow efficiency and measurement consistency; no formal economic analyses reported	Mostly internal validation; few external/multicenter studies; heterogeneous, single-center datasets limit generalizability	scattered global contributions; equity, demographic, and resource-setting impacts underreported	Data quality and heterogeneity; lack of pathological cases; real-time system integration; operator–AI interface development challenges	Underaddressed needs for model explainability, patient privacy/consent, bias mitigation, and adherence to AI reporting guidelines
Sharifi-Heris et al., 2022 (Sharifi-Heris et al., 2022)	systematic review to predict preterm birth	Predicting preterm birth risk from sociodemographic, obstetric history, clinical and laboratory EHR features	Diverse supervised models (LR, SVM, RF, ANN, GBM, etc.); reported AUCs ranged ~0.62–0.98, sensitivities 40–100%, specificities 54–94%	Demonstrated predictive potential but none reached clinical deployment; no formal economic impact assessments reported	Predominantly internal validation; only 1 study performed external validation; heterogeneity	Majority of studies (69%) based in the US; nearly half did not report race/ethnicity; equity and subgroup impacts largely unexamined	Challenges with missing data management, class imbalance, feature heterogeneity, lack of real-time EHR integration and dynamic feature extraction	Underaddressed needs for model explainability, patient privacy/informed consent safeguards, bias mitigation strategies, and adherence to AI reporting guidelines
Ranjbar et al., 2024 (Ranjbar et al., 2024)	systematic review of ML models for preeclampsia prediction;	Early- and late-onset preeclampsia prediction using maternal demographics, medical/obstetric history, labs, ultrasound findings	Elastic Net, SGB, EGBM, RF, LR, DT, SVM, NB, DNN; AUC range 0.860–0.973; metrics: accuracy, precision, recall, F1, TPR, FPR	Demonstrated high predictive accuracy for early detection; no tools reached clinical deployment and no economic analyses reported	**I**nternal validation only; PROBAST:	**S**tudies conducted in the USA, South Korea, and China; equity and subgroup performance largely unreported	Data heterogeneity and missing values, lack of real-time EHR integration, limited interpretability, workflow integration challenges	Underaddressed need for explainability, patient privacy/informed consent, bias mitigation, and adherence to SPIRIT-AI/CONSORT-AI guidelines
Michalitsi et al., 2024 (Michalitsi et al., 2024)	Systematic review of AI for predicting mode of delivery	Predicting vaginal vs cesarean delivery, VBAC success, induction outcomes; shared decision-making support	Logistic regression, random forest, gradient boosting AUC 0.745–0.932	Enhanced decision support and potential reduction in unnecessary interventions; no formal economic impact assessment reported	narrative synthesis due to heterogeneity; quality assessed with PROBAST	Studies span Turkey, Ghana, Spain, China, Bangladesh, USA, Taiwan, Jordan, Korea, Sweden, Denmark; equity and demographic impacts under-reported	Data heterogeneity and missing values, lack of standardization and real-time integration, limited interpretability, workflow integration challenges	Need for explainable AI, patient privacy/informed consent safeguards, adherence to AI reporting guidelines (e.g. SPIRIT-AI, CONSORT-AI), bias mitigation
Schouten et al., 2024 (Schouten et al., 2024)	Systematic review of 262 NICU/PICU AI studies up to Mar 28 2024	Diagnostics/prognostics (sepsis, mortality, LOS, etc.)	Supervised ML prototypes; median AUC high; calibration in 3%	No routine deployment; economic impact unassessed	97% internal, 13% external; 77% high bias risk	Global studies; equity/demographic context largely unreported	Small samples, lacking data infra, explainability, UI challenges	Limited AI guideline adherence; transparency and bias mitigation gaps
Tadepalli et al., 2025 (Tadepalli et al., 2025)	Systematic review	Morphology & biometry; gestational age; congenital defects; CTG monitoring; placental & maternal status.	ML & DL (CNNs, U-Net, Transformers); mAP 0.955; MAE 0.85 weeks (R^2 0.904);	Improved diagnostic accuracy; workflow automation (minutes→seconds);	Predominantly single center; transfer learning recall ~0.80 in African settings.	Data from Europe, India, China, Morocco, select African centers;	Data scarcity; "black-box" opacity; workflow/device integration challenges.	Needs explainability; data privacy/consent; no clear obstetric AI regulatory pathway

			Dice 0.77–0.95; AUC up to 0.996.	potential cost savings (not yet quantified).		limited low-resource representation.		
Mendizabal-Ruiz et al., 2024(Mendizabal-Ruiz et al., 2024)	Narrative review	Fertility tracking; sperm/oocyte/embryo selection; lab automation; risk screening	ML/DL (CNN, SVM); embryo AUC > 90 %; sperm precision > 98 %	↑ consistency; ↑ success; ↓ time	Retrospective; single-center	Mexico, Europe, US; limited LMIC	Data scarcity; workflow fit; cost	Privacy; bias; consent; regs lacking
Sibanda et al., 2022(Sibanda et al., 2022)	Systematic review	Remote monitoring; risk prediction	ANN, CNN, ML frameworks (mAP ~0.95; prec 0.96; rec 0.93)	Improved prediction; no cost data	Mostly conceptual; few prototypes	Predominantly Asia; low-/mid-income	Security; interoperability; infra gaps	Data privacy; consent; device intrusiveness
Olawade et al. (2025) (Olawade et al., 2025)	Narrative review	Ovarian stimulation; gamete selection; embryo annotation; QC & KPI monitoring; workflow optimization	ML dosing models; CNN/DL for image/video analysis; predictive analytics	Potential ↑ fertilization/implantation rates; workflow efficiency; reduced burdens	Lacks large-scale, multi-center RCTs; single-center, homogeneous cohorts	Mostly Western populations; equity not assessed	Dataset bias; lack of standardized protocols; infrastructure requirements	Data privacy (GDPR/HIPAA); “black-box” opacity; algorithmic bias
Bou Nassif et al. (2022)(Nassif et al., 2022)	Systematic review\AI for breast cancer detection via histopathological imaging and gene sequencing	Early detection, subtype classification, prognosis prediction using imaging (H&E slides, MRI, mammography) and genomics (gene expression)	CNNs, ANNs, SVMs, hybrid DL + ML (e.g., GA-MLP, DBN-ELM), attention-based DNNs; accuracies up to 99.8% (gene data) and 99.75% (imaging)	Substantially improved diagnostic accuracy and potential for earlier intervention; economic impact not directly assessed but automation implies cost-savings	Validation mostly on single-center or public datasets (TCGA, METABRIC, UCI-Wisconsin, DDSM); limited multi-center or prospective trials	Data drawn from US, Europe, UAE and mixed public/private cohorts; equity considerations not addressed	Dependence on proprietary/private data, class imbalance, heavy compute needs, lack of standardized protocols	Data privacy (genetic/imaging), “black-box” opacity of DL models, need for interpretability and consent frameworks
Wu et al. (2025) (Wu et al., 2025)	Systematic review covering all IVF phases	COS dosing & timing; ultrasound segmentation & follicle monitoring; trigger-day decision; oocyte/sperm analysis; embryo ploidy prediction; workflow & scheduling; patient safety; micromanipulation; social egg freezing	ML (supervised, unsupervised, RL), CNNs (U-Net variants, DeepLabV3+), KNN, RF, LGBM, XGBoost, stacking ensembles; follicle segmentation Dice 0.912/0.858; trigger PN +1.43; ICSI polar-body loc 99 %; AUC 0.68–0.91; ploidy accuracy up to 77.4 %; workflow error reduction	↑ MII oocytes; ↓ total FSH; ↑ usable blastocysts; improved embryo viability; ↑ trigger accuracy; fewer clinic visits; potential cost savings via automation	Mostly retrospective single- and multi-center cohorts; few RCTs (e.g., FSH dosing trial ongoing); limited prospective validation	Data from US, Taiwan, Europe; equity/disparities not evaluated	Data quality & standardization; dataset bias; high compute needs; integration into clinical workflows; staff training	Patient data privacy & consent; “black-box” model opacity; algorithmic bias; need for regulatory oversight
Du Y. et al. (2023)(Du et al., 2023)	Systematic review of ML-based CDSSs in pregnancy care	CDSS functions across all pregnancy stages (preconception → postpartum)	Various ML (SVM, ANN, RF, AdaBoost, etc.); majority black-box with limited post-hoc explainability	Reports positive clinical impacts in limited pilot evaluations; economic benefits not quantified	No external model validation; single-centre cohorts; generalizability untested	Predominantly single-country datasets; no equity or subgroup analyses	Gap between model development and deployment; minimal user testing; UI/UX and data-quality challenges	Sparse discussion of XAI, data bias, regulatory compliance, or ethical governance
Dimitriadis I. et al. (2022)(Dimitriadis et al., 2022)	Narrative review of AI in the embryology laboratory (sperm → blastocyst → implantation)	Automated sperm/semen analysis; oocyte assessment; pronuclear, cleavage, blastocyst evaluation; embryo witnessing; non-invasive ploidy screening	CNN, ANN, SVM, decision trees; reported accuracies up to 98.9% (polar body detection), ~91% (blastocyst classification)	Improved objectivity and QA metrics in lab workflows; clinical outcome impact not yet proven	Internal retrospective validations; no multicentre RCTs; prospective clinical studies lacking	Draws on data from multiple IVF centres; no equity or demographic subgroup analysis	Reliance on small, heterogeneous, retrospectively labeled datasets; lack of standard protocols	Data privacy and sharing constraints; limited ethical/regulatory framework
Reinhart L. et al. (2024)(Reinhart et al., 2024)	Systematic review of AI in child development monitoring	Monitoring cognitive, language, social, motor, emotional, physical development; ASD	SVM, RF, deep learning, ensemble methods; domain-specific accuracies ranged ~60–99% (highest in image-based tasks)	No studies report downstream clinical or economic outcomes	Mostly internal, retrospective validations; high risk of bias; few longitudinal or real-world tests	Predominantly US, China, India; scant analysis of equity, low-resource settings	Small/cleaned datasets; heterogeneity of tasks/data; lack of stakeholder engagement & pros. trials	Data privacy concerns; minimal discussion of governance, informed consent, or liability issues

Bulletti et al., 2024 (Bulletti et al., 2024)	Systematic review of 116 CDSAs in ART	Prognosis & counseling; clinical management; embryo assessment	ML models & calculators (e.g. mAP ≈ 0.955; varied P/R metrics)	Potential ↑ IVF success; ↓ repeat cycles	Heterogeneous methods; few external validations	Europe, US, Asia; equity seldom addressed	Lack of standardization; inconsistent validation	Data privacy; informed consent; device regulation
Davidson & Boland, 2021 (Davidson & Boland, 2021)	Systematic review of 127 AI/ML studies in pregnancy	Prenatal monitoring (fetal anomalies, placental function), preterm-birth prediction, CDSS, mHealth apps, deep phenotyping	SVM (n = 30), ANN (n = 22), regression (n = 17), RF (n = 16), DL (n = 13); typical AUCs ~0.70–0.93	Demonstrated ↑ diagnostic accuracy (e.g. FHR classification), risk stratification for preterm birth; economic impacts rarely evaluated	Mostly retrospective EHR cohorts; few external or prospective validations; heterogenous methods limit generalizability	Largely US/Europe/Asia; equity and underserved populations seldom addressed	Low clinical uptake; workflow integration and real-time data pipelines lacking	Ethical implications (privacy, consent) underexplored; need standardized governance frameworks
Hew et al., 2024 (Hew et al., 2024)	Narrative review of AI integration in IVF labs—covering QC/QA, embryo & sperm selection, data workflows, imaging and robotic tools	QC/QA automation; embryo viability prediction; sperm analysis; data standardization; robotic micromanipulation	Neural networks, deep learning (CNN, DNN), machine learning (SVM, RF); AUCs up to 0.93 for embryo viability, >90% accuracy in sperm-DNA tests	Demonstrated more consistent embryo/sperm selection, reduced manual errors, streamlined lab throughput; economic benefits implied but not yet quantified	Based on individual case studies and vendor reports; few multicenter or prospective validations; heterogeneity across labs limits broad generalizability	Examples drawn from US, Europe, Asia; review notes little focus on underserved or equity issues	Data integration with LIMS; lack of standardized datasets; "black-box" anxiety; workflow fit; upfront costs	Data privacy/security; need for algorithm transparency/explainability; informed consent; regulatory void around AI in IVF labs
Patel et al. 2024 (Patel et al., 2024)	Narrative review of AI in OB/GYN—imaging, predictive analytics, personalized care, monitoring, labor & postpartum	US & MRI plane detection; fetal biometry; preterm birth, preeclampsia, GDM risk models; IVF & oncology personalization; CTG analysis; remote monitoring	CNNs for ultrasound plane segmentation (human-level performance); envelope tracing 98% accuracy; DL heart imaging AUC 0.99; CTG models AUC 0.73–0.99; mixed RCT outcomes	Enhanced diagnostic accuracy; early complication prediction; 50% reduction in neonatal seizures with continuous CTG; workflow efficiencies; cost impacts not yet quantified	Largely retrospective and single-center studies; few prospective validations; generalizability limited by dataset heterogeneity and clinician workflow integration	Data from Europe, North America, India; equity seldom addressed—bias risks in underserved populations; telemedicine uptake variable	Data privacy/HIPAA compliance; infrastructure & EHR integration; clinician training; "black-box" interpretability; data bias; limited real-world deployment	Consent & transparency; algorithmic bias; accountability for AI-driven decisions; lack of clear regulatory pathways for OB/GYN AI tools
Mapari et al. 2024 (Mapari et al., 2024)	Narrative review of AI in maternal health—early detection, personalized care, remote monitoring, accessibility	Predictive analytics (preeclampsia, GDM, preterm birth); AI ultrasound; telemedicine/chatbots; wearables; genomics; mental health	ML/DL models (AUC ~0.89–0.99 reported in examples); automated image analysis sensitivity >90%	Early intervention; improved maternal/fetal outcomes; greater rural access; mental health support; implied cost/resource efficiencies	Mainly pilot/case reports; few prospective trials; generalizability across populations untested	Global scope with focus on low-resource/rural areas (e.g. India); equity via telehealth; bias risks in underserved groups	Data privacy/security; infrastructure & training gaps; digital divide; "black-box" interpretability	Consent & transparency; algorithmic bias/fairness; accountability frameworks; mandatory human oversight
Giaxi et al., 2025 (Giaxi et al., 2025)	Review across obstetrics & midwifery	Embryo selection; pregnancy-risk stratification; maternal/fetal monitoring; labor & neonatal outcome prediction	ML/DL (RF, XGBoost, CNN, SVM, AdaBoost) with AUCs ranging 0.65–1.00	Markedly improved diagnostic/predictive accuracy; economic impact not systematically reported	External and cross-cohort validations in multiple studies	Europe, North America, Asia, Middle East; few low-resource settings; equity rarely assessed	Variable data quality and standardization; integration into clinical workflows; limited AI expertise	Data privacy/bias concerns; lack of clear regulatory and ethical guidelines for clinical AI
Włodarczyk et al., 2021 (Włodarczyk et al., 2021)	Systematic survey of 24 studies (1994–2020) across four data domains (EHG, EHR, TVS, EMG) for preterm-birth risk prediction	Early warning of spontaneous preterm birth via analysis of physiological signals (EHG/EMG), imaging (TVS), and clinical records (EHR)	SVM/RF/CNN/LSTM/ANN:<br>• EHG RF+ADASYN AUC ≈ 0.99<br>• EHR LSTM-ensemble AUC 0.827<br>• TVS CNN+U-Net AUC 0.78<br>• EMG ANN acc ≈ 92%	Potential for earlier intervention to reduce neonatal morbidity; economic impact not yet evaluated	Predominantly single-center; few external validations; performance varies by modality	Europe, North America, Asia; lack of low-resource and equity analyses	Data heterogeneity; severe class imbalance; small n; lack of clinical integration	Data privacy/consent; absence of AI-specific clinical regulations

Bartl-Pokorny et al., 2024 (Bartl-Pokorny et al., 2024)	Systematic review (2018–2022) of ML for disease detection/prediction in infants (first year of life)	Disease detection & prediction across 12 ICD-11 categories in infants	DNNs, RF, SVM most common; metrics: accuracy, AUC-ROC, sensitivity, specificity; generally good performance	Promising for early diagnosis/intervention; no direct economic analysis	Limited external validation; most studies single-center; generalizability concerns noted	Broad, global scope but equity rarely addressed explicitly	Small datasets, lack of explainability, heterogeneity in methods/data	Explainability, transparency, liability, regulation, limited reporting on ethics
Naz et al., 2025 (Naz et al., 2025)	Systematic review & meta-analysis of AI for gestational age estimation vs. ultrasound (17 studies, 10 meta-analyzed)	Gestational age estimation from ultrasound (2D & blind sweep)	DNN, CNN, FCN; Mean error: 4.32 days (2D), 2.55 days (video); better in 2nd trimester; good accuracy	Promising accuracy, esp. for LMICs; no direct economic evaluation	Limited external validation; high heterogeneity; generalizability concerns	Mostly HIC/UMIC; few LMIC; equity noted as a gap	Data quality, limited external validation, heterogeneity	Data privacy, need for representative datasets, regulatory/ethical gaps
Khan et al., 2022<br><br>(Khan et al., 2022)	Narrative Review on AI in maternal and neonatal health in low-resource settings	Maternal monitoring, preterm birth prediction, gestational diabetes, neonatal pain, sepsis, jaundice detection	ML, DL, IoT-based systems; Models achieved AUCs ~0.8–0.9, high accuracy in various tasks	Promising early detection; no direct economic analysis; focus on reducing mortality	Mostly small datasets; single-center validations; generalizability remains limited	Focus on LMICs; equity challenges in AI access and infrastructure	Data scarcity, model bias, need for low-cost tech, trust in AI	Privacy concerns, lack of explainability, need for ethical design, regulatory gaps
Kakkar et al., 2025<br><br>(Kakkar et al., 2025)	Narrative Review; broad literature synthesis on AI in assisted reproduction	Stimulation protocol optimization, sperm/oocyte/embryo selection, live birth prediction, patient communication	CNN, DL, ML algorithms; high predictive accuracy (blastocyst formation 60–95%); good for embryo grading	Enhanced treatment personalization, diagnostic accuracy; no direct economic analysis	Mostly small datasets; evolving models; poor external validation; generalization issues	Mainly HIC data; lack of diversity noted; equity concerns discussed	Data scarcity, heterogeneity, model bias, trust, regulatory gaps	Transparency, explainability, liability issues; need for regulatory frameworks
Mills et al., 2023 (Mills et al., 2023)	Realist synthesis; chatbot use in sexual and reproductive health (SRH)	SRH education, counseling, service linkage	Mostly rule-based and NLP-driven chatbots; focus on conversational quality, responsiveness	Promising for improving SRH information access and service connection; no direct economic outcomes	Limited evaluation; mixed-quality studies; strong generalizability issues	Broad (HICs and LMICs); equity concerns in digital access and stigma contexts	Stigma, device access, chatbot limitations (e.g., unnatural conversation)	Privacy, confidentiality, trust, human oversight integration needed
Lin et al., 2024 (X et al., 2024)	Systematic Review; AI-augmented CDSS in pregnancy care	Risk prediction, diagnosis, treatment recommendation, knowledge base construction	ML models (SVM, RF, XGBoost, CNN, LSTM); generally high internal accuracy (AUCs ~0.7–0.9)	Promising early detection (e.g., GDM, preeclampsia); no direct economic evaluations	Most studies only internal validation; limited external validation; generalizability issues	Mainly HICs; limited LMIC focus; equity issues largely unaddressed	Poor external validation, bias risk, limited real-world implementation	Transparency, bias, lack of SDOH considerations, regulatory needs
Sullivan et al., 2023 (Sullivan et al., 2023)	Narrative review on AI in neonatology	Sepsis prediction, NEC diagnosis, BPD prediction, ROP detection, brain injury detection, oxygen titration	ML, DL, SVM, decision trees, neural networks; generally high internal performance	Improved early diagnosis potential; no direct economic evaluations	Mostly retrospective; limited prospective/RCT validation; generalizability gaps	Predominantly HICs; equity issues acknowledged	Data quality, small datasets, lack of external validation, clinician distrust	Bias, fairness, explainability, transparency, regulatory frameworks
Steinberg et al., 2025 (Steinberg et al., 2025)	Scoping review; ML in peripartum care (406 studies)	Risk prediction, diagnosis (fetal distress, PTB, birth weight, mode of delivery, PPH)	Supervised ML dominant (SVM, RF, LR, KNN, DT); generally good internal performance	Promising for early prediction; no direct economic evaluations	63% internal validation; only 5% external validation; generalizability concerns	Mostly HICs (US, China); LMICs underrepresented	Lack of external validation, data access, explainability, EHR integration challenges	Transparency, fairness, regulatory frameworks, decolonization of AI in global health
Ramakrishnan et al., 2021 (Ramakrishnan et al., 2021)	Narrative Review; AI in perinatal health	Prediction of PTB, LBW, birth defects, maternal morbidity, neonatal mortality	ML models (SVM, DT, ANN); success varies; some outperform traditional methods	Potential to improve early diagnosis and outcomes; no direct economic evaluations	Data gaps, small datasets; generalizability concerns noted	HICs and LICs both discussed; stressed disparities	Data availability, model explainability, clinician trust	Need for explainable AI; societal acceptance emphasized
Davidson & Boland, 2020	Systematic review on AI in pregnancy, especially	Clinical data mining, decision support systems,	ML models (RF, SVM, ANN); variable accuracy; good	Potential to improve pharmacologic safety in	Mostly retrospective data; very limited external validation	HIC focus; equity and vulnerable populations acknowledged	Data scarcity, lack of pharmacologic-specific AI models,	Transparency, explainability, regulatory needs for pregnancy-specific AI tools

(Davidson & Boland, 2020)	pharmacologic exposure	translational studies from animal to human	potential but limited prospective validation	pregnancy; no direct economic analysis			underrepresentation in trials	
Islam et al., 2022 (M. N. Islam et al., 2022)	Systematic Review; ML for pregnancy outcomes (26 studies)	Predicting mode of delivery, preterm birth, risks/complications, IVF outcomes	ML models (SVM, RF, DT, LR, NB); accuracy varied; DT, RF often highest	Improved prediction of complications and outcomes; no direct economic evaluation	Mostly small datasets; few prospective studies; generalizability concerns	Mix of HICs and LMICs; LMICs underrepresented	Data quality, lack of datasets from high-need areas, explainability issues	Data privacy, fairness, model explainability, need for better regulatory oversight
Islam et al., 2025 (S. Islam et al., 2025a)	Systematic review of AI-based risk assessment in sexual, reproductive, and mental health	Triage, symptom checkers, risk prediction tools	ML algorithms (SVM, RF, DL, NLP); variable accuracy, often outperform traditional methods	Improved prediction and triage for SRH and MH; no direct economic evaluation	Majority internal validation; poor external validation; generalizability limited	Mostly HICs; LMIC underrepresented; equity gaps highlighted	Data scarcity, model bias, lack of external validation, digital divide	Privacy, consent, fairness, need for transparent and ethical AI frameworks
Chng et al., 2025 (Chng et al., 2025)	Narrative review on AI ethics in child health; PEARL-AI framework proposed	Ethical and governance principles for AI in pediatric healthcare	Not a model evaluation; focused on ethical principles and frameworks	No clinical or economic outcome analysis (ethics focus)	Not applicable (conceptual review)	Addresses equity and justice concerns; promotes inclusivity across diverse populations	Lack of pediatric-specific AI policies; lack of pediatric datasets; underrepresentation in AI development	Extensive coverage: autonomy, beneficence, non-maleficence, justice, transparency, privacy, dependability, accountability, auditability, trust
Gulzar Ahmad et al., 2022 (Gulzar Ahmad et al., 2022)	Narrative Review; wearable sensors and AI for maternal-infant health	Maternal monitoring (BP, HR, fetal health), infant monitoring (movement, HR, temp)	ML models (SVM, ANN, RF, CNN); generally high accuracy in internal evaluations	Potential improvement in early diagnosis and maternal/infant outcomes; no direct economic evaluation	Mostly small datasets; limited external validation; generalizability concerns noted	Focus on rural vs. urban divide; equity emphasized for remote care	Data scarcity, integration challenges, reliability and power consumption issues	Privacy, data security, explainability, regulation needs
Elia Abou Chawareb et al., 2025 (Abou Chawareb et al., 2025)	Scoping Review; AI applications in sexual medicine	Diagnosis and management of STIs, infertility, sexual dysfunction, relationship issues; forensic sex determination	ML, DL, NLP, chatbots; high performance in diagnosis and prediction tasks	Improved diagnosis, early detection, and public health interventions; no direct economic evaluations	Mostly retrospective; limited external validation; generalizability concerns	Focused on HICs; LMICs underrepresented; equity and accessibility highlighted	Data quality, bias, need for personalization, user trust issues	Privacy, confidentiality, fairness, regulatory gaps, need for ethical frameworks
Rajput et al., 2024 (Amruta Rajput, 2024)	Narrative Review; AI in infertility diagnosis and treatment	Imaging (ultrasound), sperm analysis, embryo selection, treatment personalization, genetic screening	ML models (SVM, RF, CNN, DL); good internal performance; predictive analytics for IVF success	Potential for improved pregnancy outcomes, personalization, and ART optimization; no direct economic analysis	Limited external validation; emphasis on small datasets and need for clinical validation	Mainly HIC settings; equity issues in access to advanced fertility AI highlighted	Data privacy, model bias, clinician trust, integration challenges	Data security, consent, bias, fairness, regulatory oversight needed
Panda and Sharma, 2024 (Panda & Sharma, 2024)	Narrative Review; AI in maternal health, pregnancy, fertility	Prediction of complications, remote monitoring, CDSS, fertility treatment support	ML models (unspecified); promising predictive accuracy for GDM, PTB, preeclampsia	Potential for reduced maternal morbidity, earlier interventions, fertility success; no direct economic analysis	Limited external validation; most evidence early-phase; real-world integration challenges	Focus on LMICs, tribal areas; equity improvement emphasized	Data security, algorithm bias, technological infrastructure gaps	Privacy, fairness, transparency, regulation needs

Abbreviation	Definition
**AC**	Abdominal circumference
**AI**	Artificial intelligence

Abbreviation	Definition
**ANN**	Artificial neural network
**ART**	Assisted reproductive technology
**ASD**	Autism spectrum disorder
**AUC**	Area under the (receiver operating characteristic) curve
**CDSA**	Clinical decision-support algorithm/system
**CDSS**	Clinical decision-support system
**CHD**	Congenital heart defect(s)
**CNN**	Convolutional neural network
**COS**	Controlled ovarian stimulation
**CV**	Computer vision
**DBN-ELM**	Deep belief network – extreme learning machine
**DL**	Deep learning
**DNN**	Deep neural network
**DT**	Decision tree
**DSC**	Dice similarity coefficient
**DDSM**	Digital Database for Screening Mammography
**EGBM**	Extreme gradient boosting machine
**EHG**	Electrohysterography
**EMG**	Electromyography
**EHR**	Electronic health record
**FCN**	Fully convolutional network
**FHR**	Fetal heart rate
**FINE**	Fetal intelligent navigation echocardiography
**FSH**	Follicle-stimulating hormone
**FP**	False positive rate
**GA**	Gestational age
**GBM**	Gradient boosting machine
**GDM**	Gestational diabetes mellitus

Abbreviation	Definition
**HC**	Head circumference
**H&E**	Hematoxylin & eosin (staining)
**HIC**	High-income country(ies)
**ICD-11**	International Classification of Diseases, 11th Revision
**ICSI**	Intracytoplasmic sperm injection
**IoT**	Internet of Things
**KNN**	k-nearest neighbors
**LBW**	Low birth weight
**LGBM**	Light gradient boosting machine
**LIMS**	Laboratory information management system
**LMIC**	Low- and middle-income country(ies)
**LR**	Logistic regression
**LSTM**	Long short-term memory (recurrent neural network)
**LOS**	Length of stay
**MAE**	Mean absolute error
**METABRIC**	Molecular Taxonomy of Breast Cancer International Consortium
**mAP**	Mean average precision
**MRI**	Magnetic resonance imaging
**NB**	Naive Bayes
**NEC**	Necrotizing enterocolitis
**NT**	Nuchal translucency
**NLP**	Natural language processing
**PBMC**	[Removed—was not used]
**PICU**	Pediatric intensive care unit
**PN**	Pronuclear (stage)
**PPH**	Postpartum hemorrhage
**PROBAST**	Prediction model Risk Of Bias ASsessment Tool
**PRISMA**	Preferred Reporting Items for Systematic Reviews and Meta-Analyses

Abbreviation	Definition
**PTB**	Preterm birth
**QC**	Quality control
**QA**	Quality assurance
**RF**	Random forest
**RL**	Reinforcement learning
**RNN**	Recurrent neural network
**ROC**	Receiver operating characteristic
**R^2**	Coefficient of determination
**SGB**	Stochastic gradient boosting
**SDOH**	Social determinants of health
**SRH**	Sexual and reproductive health
**SVM**	Support vector machine
**TCGA**	The Cancer Genome Atlas
**TPR**	True positive rate
**TRL**	Technology readiness level
**UCI-Wisconsin**	University of California, Irvine – Wisconsin Breast Cancer Dataset
**U-Net**	Convolutional network architecture for biomedical image segmentation
**VBAC**	Vaginal birth after cesarean
**XAI**	Explainable artificial intelligence
**XGBoost**	Extreme gradient boosting
**TVS**	Transvaginal sonography

References

Abou Chawareb, E., Im, B. H., Lu, S., Hammad, M. A. M., Huang, T. R., Chen, H., & Yafi, F. A. (2025). Sexual health in the era of artificial intelligence: a scoping review of the literature. *Sexual Medicine Reviews*, *13*(2), 267–279. https://doi.org/10.1093/SXMREV/QEAF009

Amruta Rajput, S. D. , Dr. K. R. (2024). The Role of Artificial Intelligence in Revolutionizing Infertility Diagnosis and Treatment: Current Applications and Future Directions. *International Journal of Pharmaceutical Sciences*, *2*, 1413–1422. https://doi.org/10.5281/ZENODO.14223718

Arab, R. A. El, Abu-Mahfouz, M. S., Abuadas, F. H., Alzghoul, H., Almari, M., Ghannam, A., & Seweid, M. M. (2025). Bridging the Gap: From AI Success in Clinical Trials to Real-World Healthcare Implementation—A Narrative Review. *Healthcare 2025, Vol. 13, Page 701*, *13*(7), 701. https://doi.org/10.3390/HEALTHCARE13070701

Baethge, C., Goldbeck-Wood, S., & Mertens, S. (2019). SANRA—a scale for the quality assessment of narrative review articles. *Research Integrity and Peer Review*, *4*(1), 1–7. https://doi.org/10.1186/S41073-019-0064-8/TABLES/1

Bartl-Pokorny, K. D., Zitta, C., Beirit, M., Vogrinec, G., Schuller, B. W., & Pokorny, F. B. (2024). Focused review on artificial intelligence for disease detection in infants. *Frontiers in Digital Health*, *6*, 1459640. https://doi.org/10.3389/FDGTH.2024.1459640/BIBTEX

Bulletti, C., Franasiak, J. M., Busnelli, A., Sciorio, R., Berrettini, M., Aghajanova, L., Bulletti, F. M., & Ata, B. (2024). Artificial Intelligence, Clinical Decision Support Algorithms, Mathematical Models, Calculators Applications in Infertility: Systematic Review and Hands-On Digital Applications. *Mayo Clinic Proceedings: Digital Health*, *2*(4), 518–532. https://doi.org/10.1016/J.MCPDIG.2024.08.007

Chng, S. Y., Tern, M. J. W., Lee, Y. S., Cheng, L. T. E., Kapur, J., Eriksson, J. G., Chong, Y. S., & Savulescu, J. (2025). Ethical considerations in AI for child health and recommendations for child-centered medical AI. *Npj Digital Medicine 2025 8:1*, *8*(1), 1–10. https://doi.org/10.1038/s41746-025-01541-1

Choi, G. J., & Kang, H. (2022). Introduction to Umbrella Reviews as a Useful Evidence-Based Practice. *Journal of Lipid and Atherosclerosis*, *12*(1), 3. https://doi.org/10.12997/JLA.2023.12.1.3

Davidson, L., & Boland, M. R. (2020). Enabling pregnant women and their physicians to make informed medication decisions using artificial intelligence. *Journal of Pharmacokinetics and Pharmacodynamics*, *47*(4), 305–318. https://doi.org/10.1007/S10928-020-09685-1/FIGURES/2

Davidson, L., & Boland, M. R. (2021). Towards deep phenotyping pregnancy: a systematic review on artificial intelligence and machine learning methods to improve pregnancy outcomes. *Briefings in Bioinformatics*, *22*(5), 1–29. https://doi.org/10.1093/BIB/BBAA369

Dimitriadis, I., Zaninovic, N., Badiola, A. C., & Bormann, C. L. (2022). Artificial intelligence in the embryology laboratory: a review. *Reproductive Biomedicine Online*, *44*(3), 435–448. https://doi.org/10.1016/J.RBMO.2021.11.003

Du, Y., McNestry, C., Wei, L., Antoniadi, A. M., McAuliffe, F. M., & Mooney, C. (2023). Machine learning-based clinical decision support systems for pregnancy care: A systematic review. *International Journal of Medical Informatics*, *173*. https://doi.org/10.1016/J.IJMEDINF.2023.105040

El Arab, R. A., & Al Moosa, O. A. (2025). The role of AI in emergency department triage: An integrative systematic review. *Intensive and Critical Care Nursing*, *89*, 104058. https://doi.org/10.1016/J.ICCN.2025.104058

El Arab, R. A., Al Moosa, O. A., Abuadas, F. H., & Somerville, J. (2025). The Role of Artificial Intelligence in Nursing Education, and Practice: An Umbrella Review. *Journal of Medical Internet Research*. https://doi.org/10.2196/69881

El Arab, R. A., Alkhunaizi, M., Alhashem, Y. N., Al Khatib, A., Bubsheet, M., & Hassanein, S. (2025). Artificial intelligence in vaccine research and development: an umbrella review. *Frontiers in Immunology*, *16*, 1567116. https://doi.org/10.3389/FIMMU.2025.1567116/BIBTEX

El Arab, R. A., Almoosa, Z., Alkhunaizi, M., Abuadas, F. H., & Somerville, J. (2025). Artificial intelligence in hospital infection prevention: an integrative review. *Frontiers in Public Health*, *13*, 1547450. https://doi.org/10.3389/FPUBH.2025.1547450/BIBTEX

Giaxi, P., Vivilaki, V., Sarella, A., Harizopoulou, V., & Gourounti, K. (2025). Artificial Intelligence and Machine Learning: An Updated Systematic Review of Their Role in Obstetrics and Midwifery. *Cureus*, *17*(3). https://doi.org/10.7759/CUREUS.80394

Gulzar Ahmad, S., Iqbal, T., Javaid, A., Ullah Munir, E., Kirn, N., Ullah Jan, S., & Ramzan, N. (2022). Sensing and Artificial Intelligent Maternal-Infant Health Care Systems: A Review. *Sensors (Basel, Switzerland)*, *22*(12). https://doi.org/10.3390/S22124362

Hassanein, S., El Arab, R. A., Abdrbo, A., Abu-Mahfouz, M. S., Gaballah, M. K. F., Seweid, M. M., Almari, M., & Alzghoul, H. (2025). Artificial intelligence in nursing: an integrative review of clinical and operational impacts. *Frontiers in Digital Health*, *7*, 1552372. https://doi.org/10.3389/FDGTH.2025.1552372

Hew, Y., Kutuk, D., Duzcu, T., Ergun, Y., & Basar, M. (2024). Artificial Intelligence in IVF Laboratories: Elevating Outcomes Through Precision and Efficiency. *Biology 2024, Vol. 13, Page 988*, *13*(12), 988. https://doi.org/10.3390/BIOLOGY13120988

Islam, M. N., Mustafina, S. N., Mahmud, T., & Khan, N. I. (2022). Machine learning to predict pregnancy outcomes: a systematic review, synthesizing framework and future research agenda. *BMC Pregnancy and Childbirth*, *22*(1), 1–19. https://doi.org/10.1186/S12884-022-04594-2/FIGURES/8

Islam, S., Shahriyar, R., Agarwala, A., Zaman, M., Ahamed, S., Rahman, R., Chowdhury, M. H., Sarker, F., & Mamun, K. A. (2025a). Artificial intelligence-based risk assessment tools for sexual, reproductive and mental health: a systematic review. *BMC Medical Informatics and Decision Making*, *25*(1), 1–25. https://doi.org/10.1186/S12911-025-02864-5/FIGURES/9

Islam, S., Shahriyar, R., Agarwala, A., Zaman, M., Ahamed, S., Rahman, R., Chowdhury, M. H., Sarker, F., & Mamun, K. A. (2025b). Artificial intelligence-based risk assessment tools for sexual, reproductive and mental health: a systematic review. *BMC Medical Informatics and Decision Making*, *25*(1), 1–25. https://doi.org/10.1186/S12911-025-02864-5/FIGURES/9

Kakkar, P., Gupta, S., Paschopoulou, K. I., Paschopoulos, I., Paschopoulos, I., Siafaka, V., & Tsonis, O. (2025). The integration of artificial intelligence in assisted reproduction: a comprehensive review. *Frontiers in Reproductive Health*, *7*, 1520919. https://doi.org/10.3389/FRPH.2025.1520919/BIBTEX

Khan, M., Khurshid, M., Vatsa, M., Singh, R., Duggal, M., & Singh, K. (2022). On AI Approaches for Promoting Maternal and Neonatal Health in Low Resource Settings: A Review. *Frontiers in Public Health*, *10*, 880034. https://doi.org/10.3389/FPUBH.2022.880034/BIBTEX

Mapari, S. A., Shrivastava, D., Dave, A., Bedi, G. N., Gupta, A., Sachani, P., Kasat, P. R., & Pradeep, U. (2024). Revolutionizing Maternal Health: The Role of Artificial Intelligence in Enhancing Care and Accessibility. *Cureus*, *16*(9), e69555. https://doi.org/10.7759/CUREUS.69555

Mendizabal-Ruiz, G., Paredes, O., Álvarez, Á., Acosta-Gómez, F., Hernández-Morales, E., González-Sandoval, J., Mendez-Zavala, C., Borrayo, E., & Chavez-Badiola, A. (2024). Artificial Intelligence in Human Reproduction. *Archives of Medical Research*, *55*(8), 103131. https://doi.org/10.1016/J.ARCMED.2024.103131

Methley, A. M., Campbell, S., Chew-Graham, C., McNally, R., & Cheraghi-Sohi, S. (2014). PICO, PICOS and SPIDER: A comparison study of specificity and sensitivity in three search tools for qualitative systematic reviews. *BMC Health Services Research*, *14*(1), 1–10. https://doi.org/10.1186/S12913-014-0579-0/TABLES/7

Michalitsi, K., Metallinou, D., Diamanti, A., Georgakopoulou, V. E., Kagkouras, I., Tsoukala, E., & Sarantaki, A. (2024). Artificial Intelligence in Predicting the Mode of Delivery: A Systematic Review. *Cureus*, *16*(9). https://doi.org/10.7759/CUREUS.69115

Mills, R., Mangone, E. R., Lesh, N., Mohan, D., & Baraitser, P. (2023). Chatbots to Improve Sexual and Reproductive Health: Realist Synthesis. *J Med Internet Res 2023;25:E46761 Https://Www.Jmir.Org/2023/1/E46761*, *25*(1), e46761. https://doi.org/10.2196/46761

Montgomery-Csobán, T., Kavanagh, K., Murray, P., Robertson, C., Barry, S. J. E., Vivian Ukah, U., Payne, B. A., Nicolaides, K. H., Syngelaki, A., Ionescu, O., Akolekar, R., Hutcheon, J. A., Magee, L. A., von Dadelszen, P., Brown, M. A., Davis, G. K., Parker, C., Walters, B. N., Sass, N., … Widmer, M. (2024). Machine learning-enabled maternal risk assessment for women with pre-eclampsia (the PIERS-ML model): a modelling study. *The Lancet Digital Health*, *6*(4), e238–e250. https://doi.org/10.1016/S2589-7500(23)00267-4,

Nassif, A. B., Talib, M. A., Nasir, Q., Afadar, Y., & Elgendy, O. (2022). Breast cancer detection using artificial intelligence techniques: A systematic literature review. *Artificial Intelligence in Medicine*, *127*, 102276. https://doi.org/10.1016/J.ARTMED.2022.102276

Naz, S., Noorani, S., Jaffar Zaidi, S. A., Rahman, A. R., Sattar, S., Das, J. K., & Hoodbhoy, Z. (2025). Use of artificial intelligence for gestational age estimation: a systematic review and meta-analysis. *Frontiers in Global Women's Health*, *6*, 1447579. https://doi.org/10.3389/FGWH.2025.1447579/BIBTEX

Olawade, D. B., Teke, J., Adeleye, K. K., Weerasinghe, K., Maidoki, M., & Clement David-Olawade, A. (2025). Artificial intelligence in in-vitro fertilization (IVF): A new era of precision and personalization in fertility treatments. *Journal of Gynecology Obstetrics and Human Reproduction*, *54*(3). https://doi.org/10.1016/J.JOGOH.2024.102903

Ouzzani, M., Hammady, H., Fedorowicz, Z., & Elmagarmid, A. (2016). Rayyan-a web and mobile app for systematic reviews. *Systematic Reviews*, *5*(1), 1–10. https://doi.org/10.1186/S13643-016-0384-4/FIGURES/6

Page, M. J., McKenzie, J. E., Bossuyt, P. M., Boutron, I., Hoffmann, T. C., Mulrow, C. D., Shamseer, L., Tetzlaff, J. M., & Moher, D. (2021). Updating guidance for reporting systematic reviews: development of the PRISMA 2020 statement. *Journal of Clinical Epidemiology*, *134*, 103–112. https://doi.org/10.1016/J.JCLINEPI.2021.02.003

Panda, P. K., & Sharma, R. (2024). Transforming maternal healthcare: Harnessing the power of artificial intelligence for improved outcomes and access. *Https://Wjarr.Com/Sites/Default/Files/WJARR-2024-2005.Pdf*, *23*(1), 662–666. https://doi.org/10.30574/WJARR.2024.23.1.2005

Panteli, D., Adib, K., Buttigieg, S., Goiana-da-Silva, F., Ladewig, K., Azzopardi-Muscat, N., Figueras, J., Novillo-Ortiz, D., & McKee, M. (2025). Artificial intelligence in public health: promises, challenges, and an agenda for policy makers and public health institutions. *The Lancet Public Health*, *10*(5). https://doi.org/10.1016/S2468-2667(25)00036-2,

Patel, D. J., Chaudhari, K., Acharya, N., Shrivastava, D., & Muneeba, S. (2024). Artificial Intelligence in Obstetrics and Gynecology: Transforming Care and Outcomes. *Cureus*, *16*(7). https://doi.org/10.7759/CUREUS.64725

Peters, M. D. J., Marnie, C., Colquhoun, H., Garritty, C. M., Hempel, S., Horsley, T., Langlois, E. V., Lillie, E., O'Brien, K. K., Tunçalp, Özge, Wilson, M. G., Zarin, W., & Tricco, A. C. (2021). Scoping reviews: reinforcing and advancing the methodology and application. *Systematic Reviews*, *10*(1), 1–6. https://doi.org/10.1186/S13643-021-01821-3/PEER-REVIEW

Ramakrishnan, R., Rao, S., & He, J. R. (2021). Perinatal health predictors using artificial intelligence: A review. *Women's Health (London, England)*, *17*. https://doi.org/10.1177/17455065211046132

Ranjbar, A., Montazeri, F., Ghamsari, S. R., Mehrnoush, V., Roozbeh, N., & Darsareh, F. (2024). Machine learning models for predicting preeclampsia: a systematic review. *BMC Pregnancy and Childbirth*, *24*(1), 1–6. https://doi.org/10.1186/S12884-023-06220-1/TABLES/2

Reinhart, L., Bischops, A. C., Kerth, J. L., Hagemeister, M., Heinrichs, B., Eickhoff, S. B., Dukart, J., Konrad, K., Mayatepek, E., & Meissner, T. (2024). Artificial intelligence in child development monitoring: A systematic review on usage, outcomes and acceptance. *Intelligence-Based Medicine*, *9*, 100134. https://doi.org/10.1016/J.IBMED.2024.100134

Schaekermann, M., Spitz, T., Pyles, M., Cole-Lewis, H., Wulczyn, E., Pfohl, S. R., Martin, D., Jaroensri, R., Keeling, G., Liu, Y., Farquhar, S., Xue, Q., Lester, J., Hughes, C., Strachan, P., Tan, F., Bui, P., Mermel, C. H., Peng, L. H., … Cameron Chen, P. H. (2024). Health equity assessment of machine learning performance (HEAL): a framework and dermatology AI model case study. *EClinicalMedicine*, *70*, 102479. https://doi.org/10.1016/j.eclinm.2024.102479

Schouten, J. S., Kalden, M. A. C. M., van Twist, E., Reiss, I. K. M., Gommers, D. A. M. P. J., van Genderen, M. E., & Taal, H. R. (2024). From bytes to bedside: a systematic review on the use and readiness of artificial intelligence in the neonatal and pediatric intensive care unit. *Intensive Care Medicine*, *50*(11). https://doi.org/10.1007/S00134-024-07629-8,

Sharifi-Heris, Z., Laitala, J., Airola, A., Rahmani, A. M., & Bender, M. (2022). Machine Learning Approach for Preterm Birth Prediction Using Health Records: Systematic Review. *JMIR Medical Informatics*, *10*(4). https://doi.org/10.2196/33875,

Shea, B. J., Reeves, B. C., Wells, G., Thuku, M., Hamel, C., Moran, J., Moher, D., Tugwell, P., Welch, V., Kristjansson, E., & Henry, D. A. (2017). AMSTAR 2: a critical appraisal tool for systematic reviews that include randomised or non-randomised studies of healthcare interventions, or both. *BMJ*, *358*. https://doi.org/10.1136/BMJ.J4008

Sibanda, K., Ndayizigamiye, P., & Twinomurinzi, H. (2022). nIndustry 4.0 Technologies in Maternal Healthcare: A Systematic Review. *IFAC-PapersOnLine*, *55*(10), 2407–2412. https://doi.org/10.1016/J.IFACOL.2022.10.069

Steinberg, S., Wong, M., Zimlichman, E., & Tsur, A. (2025). Novel machine learning applications in peripartum care: a scoping review. *American Journal of Obstetrics & Gynecology MFM*, *7*(3). https://doi.org/10.1016/J.AJOGMF.2025.101612

Sullivan, B. A., Beam, K., Vesoulis, Z. A., Aziz, K. B., Husain, A. N., Knake, L. A., Moreira, A. G., Hooven, T. A., Weiss, E. M., Carr, N. R., El-Ferzli, G. T., Patel, R. M., Simek, K. A., Hernandez, A. J., Barry, J. S., & McAdams, R. M. (2023). Transforming neonatal care with artificial intelligence: challenges, ethical consideration, and opportunities. *Journal of Perinatology 2023 44:1*, *44*(1), 1–11. https://doi.org/10.1038/s41372-023-01848-5

Tadepalli, K., Das, A., Meena, T., & Roy, S. (2025). Bridging gaps in artificial intelligence adoption for maternal-fetal and obstetric care: Unveiling transformative capabilities and challenges. *Computer Methods and Programs in Biomedicine*, *263*, 108682. https://doi.org/10.1016/J.CMPB.2025.108682

Thomas, J., & Harden, A. (2008). Methods for the thematic synthesis of qualitative research in systematic reviews. *BMC Medical Research Methodology*, *8*. https://doi.org/10.1186/1471-2288-8-45

Whiting, P., Savović, J., Higgins, J. P. T., Caldwell, D. M., Reeves, B. C., Shea, B., Davies, P., Kleijnen, J., & Churchill, R. (2016). ROBIS: A new tool to assess risk of bias in systematic reviews was developed. *Journal of Clinical Epidemiology*, *69*, 225. https://doi.org/10.1016/J.JCLINEPI.2015.06.005

Włodarczyk, T., Płotka, S., Szczepański, T., Rokita, P., Sochacki-Wójcicka, N., Wójcicki, J., Lipa, M., & Trzciński, T. (2021). Machine Learning Methods for Preterm Birth Prediction: A Review. *Electronics 2021, Vol. 10, Page 586*, *10*(5), 586. https://doi.org/10.3390/ELECTRONICS10050586

Wu, Y. C., Chia-Yu Su, E., Hou, J. H., Lin, C. J., Lin, K. B., & Chen, C. H. (2025). Artificial intelligence and assisted reproductive technology: A comprehensive systematic review. *Taiwanese Journal of Obstetrics and Gynecology*, *64*(1), 11–26. https://doi.org/10.1016/J.TJOG.2024.10.001

X, L., C, L., J, L., T, L., N, G., & B, C. (2024). Artificial Intelligence-Augmented Clinical Decision Support Systems for Pregnancy Care: Systematic Review. *Journal of Medical Internet Research*, *26*. https://doi.org/10.2196/54737

Xiao, H., Rosen, A., Chhibbar, P., Moise, L., & Das, J. (2023). From bench to bedside via bytes: Multi-omic immunoprofiling and integration using machine learning and network approaches. *Human Vaccines & Immunotherapeutics*, *19*(3), 2282803. https://doi.org/10.1080/21645515.2023.2282803

Xiao, S., Zhang, J., Zhu, Y., Zhang, Z., Cao, H., Xie, M., & Zhang, L. (2023). Application and Progress of Artificial Intelligence in Fetal Ultrasound. *Journal of Clinical Medicine 2023, Vol. 12, Page 3298*, *12*(9), 3298. https://doi.org/10.3390/JCM12093298

Yaseen, I., & Rather, R. A. (2024). A Theoretical Exploration of Artificial Intelligence's Impact on Feto-Maternal Health from Conception to Delivery. *International Journal of Women's Health*, *16*, 903. https://doi.org/10.2147/IJWH.S454127

Supplementary Table S2: AMSTAR 2(A Measurement Tool to Assess Systematic Reviews 2)

AMSTAR 2 Item	Tadepalli et al., 2025	Mendizabal-Ruiz et al., 2024	Sibanda et al., 2022	Olawade et al., 2025	Bou Nassif et al., 2022	Wu et al., 2025	Du et al., 2023	Dimitriadis et al., 2022	Reinhart et al., 2024	Bulletti et al., 2024	Davidson and Boland, 2021	Hew et al., 2024
PICO components	Yes	No	No	No	Partial Yes	Yes	Partial Yes	No	Yes	Yes	Partial Yes	No
Protocol reported	No	No	No	No	No	No	No	No	No	Yes	No	No
Study design explained	Yes	No	Partial Yes	Partial Yes	Yes	Yes	Yes	No	Yes	Yes	Yes	No
Comprehensive search	Yes	No	Yes	Yes	Yes	Yes	Yes	No	Yes	Yes	Yes	No
Study selection in duplicate	No	NA	No	No	No	No	Yes	NA	Yes	Yes	Partial Yes	NA
Data extraction in duplicate	No	NA	No	No	No	No	Yes	NA	Yes	Yes	No	NA
Excluded studies listed	Partial Yes	NA	No	No	No	No	No	NA	Yes	Partial Yes	No	NA
Included studies detailed	Yes	Partial Yes	Partial Yes	Yes	Yes	Yes	Yes	Partial Yes	Yes	Yes	Yes	Partial Yes
Risk of bias in included studies assessed	No	No	No	No	No	No	No	No	Yes	PartialYes	No	No
Sources of funding reported	No	No	No	No	No	No	No	No	No	No	No	No
Meta-analysis methods appropriate	NA	NA	NA	NA	NA	NA	NA	NA	NA	NA	NA	NA
Risk of bias impact assessed	NA	NA	NA	NA	NA	NA	NA	NA	NA	NA	NA	NA

Risk of bias accounted for in interpretation	No	No	No	No	No	No	No	No	Partial Yes	Partial Yes	No	No
Heterogeneity explained	Partial Yes	Partial Yes	Partial Yes	Partial Yes	Partial Yes	Partial Yes	Partial Yes	NA	Partial Yes	Partial Yes	Partial Yes	NA
Publication bias investigated	No	NA	No	No	No	No	No	NA	No	No	No	NA
Conflict of interest reported	Yes	Yes	Yes	Yes	Yes	Yes	Yes	Yes	Yes	Yes	Yes	Yes

AMSTAR 2 Item	Patel et al., 2024	Mapari et al., 2024	Giaxi et al., 2025	Włodarczyk et al., 2021	Bartl-Pokorny et al., 2024	Naz et al., 2025	Khan et al., 2022	Kakkar et al., 2025	Mills et al., 2023	Lin et al., 2024	Sullivan et al., 2024	Steinberg et al., 2025
PICO components	No	No	Yes	No	Partial Yes	Yes	No	No	No	Yes	No	Yes
Protocol reported	No	No	No	No	No	Yes	No	No	No	No	No	Yes
Study design explained	No	No	Yes	No	Yes	Yes	No	Partial yes	Yes	Yes	No	Yes
Comprehensive search	No	No	Yes	No	Yes	Yes	No	Partial Yes	Partial Yes	Yes	No	Yes
Study selection in duplicate	NA	NA	Yes	NA	Yes	Yes	NA	NA	No	Yes	NA	Yes
Data extraction in duplicate	NA	NA	Yes	NA	Yes	Yes	NA	NA	No	Yes	NA	Yes
Excluded studies listed	NA	NA	Yes	NA	No	Yes	No	No	No	Yes	NA	Yes
Included studies detailed	Partial Yes	Partial Yes	Yes	Partial Yes	Yes	Yes	Partial Yes	Partial yes	Partial Yes	Yes	Partial Yes	Yes
Risk of bias in included	No	No	No	No	No	Yes	No	No	No	No	No	No

studies assessed												
Sources of funding reported	No	No	No	No	No	No	No	No	No	NA	No	No
Meta-analysis methods appropriate	NA	NA	NA	NA	NA	Yes	NA	NA	NA	NA	NA	NA
Risk of bias impact assessed	NA	NA	NA	NA	NA	Yes	NA	NA	NA	NA	NA	NA
Risk of bias accounted for in interpretation	No	No	No	No	No	Yes	No	No	No	Partial Yes	No	No
Heterogeneity explained	NA	NA	Partial Yes	NA	Partial Yes	Yes	NA	Partial yes	NA	Partial Yes	NA	Partial Yes
Publication bias investigated	NA	NA	No	NA	No	No	No	No	No	No	No	No
Conflict of interest reported	Yes	Yes	Yes	Yes	Yes	Yes	Yes	Yes	Yes	Yes	Yes	Yes

AMSTAR 2 Item	Ramakrishnan et al., 2021	Davidson & Boland, 2020	Islam et al., 2022	Islam et al., 2025	Chng et al., 2025	Ahmad et al., 2022	Chawareb et al., 2025	Rajput et al., 2024	Panda & Sharma, 2024	Schouten et al. (2024)	Michalitsi et al. (2024)	Ranjbar et al. (2024)
PICO components	No	Yes	Yes	Yes	No	No	No	No	No	Yes	Yes	Yes
Protocol reported	No	No	No	No	No	No	No	No	No	Yes	No	NO
Study design explained	No	Yes	Yes	Yes	Yes	Yes	Yes	Yes	Yes	Yes	Yes	Yes
Comprehensive search	No	Yes	Yes	Yes	Yes	No	Yes	No	No	Yes	Yes	Yes

Study selection in duplicate	NA	NA	No	Yes	No	No	Yes	No	No	Yes	Yes	Yes
Data extraction in duplicate	NA	NA	No	Yes	No	No	Yes	No	No	Yes	No	Yes
Excluded studies listed	No	No	No	No	No	No	No	No	No	No	No	No
Included studies detailed	Partial Yes	Partial Yes	yes	Yes	No	Yes	Yes	Partial Yes	Partial Yes	Yes	Yes	Yes
Risk of bias in included studies assessed	No	No	No	Yes	No	No	No	No	No	Yes	Yes	Yes
Sources of funding reported	No	No	No	No	No	No	No	No	No	No	No	No
Meta-analysis methods appropriate	NA	NA	NA	NA	NA	NA	NA	NA	NA	NA	NA	NA
Risk of bias impact assessed	NA	NA	NA	NA	NA	NA	NA	NA	NA	NA	NA	NA
Risk of bias accounted for in interpretation	No	No	No	yes	No	No	No	No	No	Yes	Yes	Yes
Heterogeneity explained	NA	NA	Partial Yes	Partial Yes	NA	No	Partial yes	No	No	Yes	Yes	Yes
Publication bias investigated	No	No	No	No	No	No	No	No	No	No	No	No
Conflict of interest reported	Yes	Yes	Yes	Yes	Yes	Yes	Yes	Yes	Yes	Yes	Yes	Yes

AMSTAR 2 Item	Sharifi-Heris et al. (2022)	Yaseen & Rather (2024)	Islam et al., 2022
PICO components	Yes	No	No

Protocol reported	No	No	No
Study design explained	Yes	No	No
Comprehensive search	Yes	No	No
Study selection in duplicate	Yes	No	No
Data extraction in duplicate	Yes	No	No
Excluded studies listed	No	No	No
Included studies detailed	yes	No	No
Risk of bias in included studies assessed	No	No	No
Sources of funding reported	No	Yes	NA
Meta-analysis methods appropriate	NA	NA	NA
Risk of bias impact assessed	NA	NA	NA
Risk of bias accounted for in interpretation	Yes	No	No
Heterogeneity explained	Yes	No	No
Publication bias investigated	No	No	No
Conflict of interest reported	Yes	Yes	Yes

Supplementary Table S3A: ROBIS

Study	D1: Eligibility Criteria	D2: Study Selection	D3: Data Collection and Appraisal	D4: Synthesis and Findings	Overall ROBIS Judgment
Schouten et al. (2024)	Low	Low	Low	Low	Low
Michalitsi et al. (2024)	High	Low	High	Low	High
Ranjbar et al. (2024)	High	Low	Low	Low	Moderate
Sharifi-Heris et al. (2022)	High	Low	Low	High	High
Tadepalli et al., 2025	Low	Low	High	unclear	High
Sibanda et al., 2022	Low	Low	High	Unclear	High
Bou Nassif et al., 2022	Low	Unclear	High	Unclear	High
Wu et al., 2025	Low	Low	High	Unclear	High
Du et al., 2023	Low	Low	High	Unclear	High
Reinhart et al., 2024	Low	Low	Low	Unclear	Unclear
Bulletti et al., 2024	Low	Low	High	Unclear	High
Davidson & Boland, 2021	Low	Low	High	Unclear	High
Giaxi et al., 2025	Low	Low	High	Unclear	High

Bartl-Pokorny et al., 2024	Low	Low	High	Unclear	High
Naz et al., 2025	Low	Low	Low	Low	Low
Lin et al., 2024	Low	Low	High	Unclear	High
Davidson & Boland, 2020	Low	Low	High	Unclear	High
Islam et al., 2022	Low	Low	High	Unclear	High
Islam et al., 2025	Low	Low	Low	Unclear	Unclear
Ahmad et al., 2022	Unclear	Low	High	Unclear	High

Supplementary Table S3B: SANARA

Study	Item 1: Justification of Article Type	Item 2: Statement of Aims	Item 3: Description of Literature Search	Item 4: Referencing	Item 5: Scientific Reasoning	Item 6: Presentation of Evidence	Overall SANRA Score (0\12)
Yaseen & Rather (2024)	2/2	2/2	0/2	2/2	2/2	1/2	9/12
Xiao et al. (2023)	2/2	2/2	0/2	2/2	2/2	2/2	10/12
Mendizabal-Ruiz G et al., 2024	1/2	2/2	0/2	2/2	2/2	1/2	8/12
Olawade et al., 2025	2/2	2/2	2/2	2/2	2/2	2/2	12/12
Dimitriadis et al., 2022	1/2	2/2	0/2	2/2	2/2	2/2	9/12
Hew et al., 2024	1/2	2/2	0/2	2/2	2/2	2/2	9/12
Patel et al., 2024	1/2	2/2	0/2	2/2	2/2	2/2	9/12
Mapari et al., 2024	1/2	2/2	0/2	2/2	2/2	2/2	9/12
Włodarczyk et al., 2021	1/2	2/2	0/2	2/2	2/2	2/2	9/12
Khan et al., 2022	1/2	2/2	0/2	2/2	2/2	2/2	9/12
Kakkar et al., 2025	1/2	2/2	1/2	2/2	2/2	2/2	10/12
Mills et al., 2023	2/2	2/2	2/2	2/2	2/2	2/2	12/12
Sullivan et al., 2024	1/2	2/2	0/2	2/2	2/2	2/2	9/12

Ramakrishnan et al., 2021	1/2	2/2	0/2	2/2	2/2	1/2	8/12
Chng et al., 2025	1/2	2/2	2/2	2/2	2/2	2/2	11/2
Rajput et al., 2024	1/2	2/2	0/2	2/2	2/2	2/2	9/12
Panda & Sharma, 2024	1/2	2/2	0/2	2/2	2/2	2/2	9/12

Supplementary Table S3C: Scoping JBI

Study	Appraisal Tool	Implementation Notes
Steinberg et al., 2025	JBI Scoping Review Guidance (no RoB tool)	As per JBI best practice for scoping reviews—and in line with the authors' own "no formal quality assessment" approach—no standardized risk-of-bias assessment was performed.
Chawareb et al., 2025	JBI Scoping Review Guidance (no RoB tool)	In line with JBI recommendations for scoping reviews, no formal risk-of-bias assessment was conducted; the authors transparently reported their eligibility criteria, search strategy, and selection process.
